# Impact of preconception and antenatal supplementation with *myo*-inositol, probiotics, and micronutrients on offspring BMI and weight gain over the first 2 years

**DOI:** 10.1186/s12916-024-03246-w

**Published:** 2024-01-30

**Authors:** Jaz Lyons-Reid, José G. B. Derraik, Timothy Kenealy, Benjamin B. Albert, J. Manuel Ramos Nieves, Cathriona R. Monnard, Phil Titcombe, Heidi Nield, Sheila J. Barton, Sarah El-Heis, Elizabeth Tham, Keith M. Godfrey, Shiao-Yng Chan, Wayne S. Cutfield, NiPPeR Study Group, Ryan Carvalho, Julie Ann Castro, Mary Cavanagh, Hsin Fang Chang, Yap Seng Chong, Paula Costello, Vanessa Cox, Sevasti Galani, Judith Hammond, Nicholas C. Harvey, Soo Min Han, Mrunalini Jagtap, Chiara Nembrini, Justin M. O’Sullivan, Judith Ong, Irma Silva-Zolezzi, Wendy Sim, Vicky Tay, Mya-Thway Tint, Mark Vickers, Jui-Tsung Wong, Gladys Woon, Wen Lun Yuan

**Affiliations:** 1 Liggins Institute, The University of Auckland, Private Bag 92019, Auckland, New Zealand; 2 Department of Paediatrics: Child and Youth Health, Faculty of Medical and Health Sciences, The University of Auckland, Auckland, New Zealand; 3 Environmental-Occupational Health Sciences and Non-Communicable Diseases Research Group, Research Institute for Health Sciences, Chiang Mai University, Chiang Mai, Thailand; 4 Department of Women’s and Children’s Health, Uppsala University, Uppsala, Sweden; 5 Department of Medicine and Department of General Practice and Primary Health Care, The University of Auckland, Auckland, New Zealand; 6 Nestlé Institute of Health Sciences, Nestlé Research, Société Des Produits Nestlé S.A, Lausanne, Switzerland; 7 MRC Lifecourse Epidemiology Centre, University of Southampton, Southampton, UK; 8 NIHR Southampton Biomedical Research Centre, University of Southampton and University Hospital Southampton NHS Foundation Trust, Southampton, UK; 9 Singapore Institute for Clinical Sciences, Agency for Science, Technology and Research (A*STAR), Singapore, Singapore; 10 Human Potential Translational Research Programme, Yong Loo Lin School of Medicine, National University of Singapore, Singapore, Singapore; 11 Department of Obstetrics & Gynaecology, National University of Singapore, Singapore, Singapore; 12 A Better Start – National Science Challenge, The University of Auckland, Auckland, New Zealand

**Keywords:** Nutritional supplementation, Infant weight gain, Preconception, Pregnancy, Randomised trial

## Abstract

**Background:**

Nutritional intervention preconception and throughout pregnancy has been proposed as an approach to promoting healthy postnatal weight gain in the offspring but few randomised trials have examined this.

**Methods:**

Measurements of weight and length were obtained at multiple time points from birth to 2 years among 576 offspring of women randomised to receive preconception and antenatally either a supplement containing *myo*-inositol, probiotics, and additional micronutrients (intervention) or a standard micronutrient supplement (control). We examined the influence on age- and sex-standardised BMI at 2 years (WHO standards, adjusting for study site, sex, maternal parity, smoking and pre-pregnancy BMI, and gestational age), together with the change in weight, length, BMI from birth, and weight gain trajectories using latent class growth analysis.

**Results:**

At 2 years, there was a trend towards lower mean BMI among intervention offspring (adjusted mean difference [aMD] − 0.14 SD [95% CI 0.30, 0.02], *p* = 0.09), and fewer had a BMI > 95th percentile (i.e. > 1.65 SD, 9.2% vs 18.0%, adjusted risk ratio [aRR] 0.51 [95% CI 0.31, 0.82], *p* = 0.006). Longitudinal data revealed that intervention offspring had a 24% reduced risk of experiencing rapid weight gain > 0.67 SD in the first year of life (21.9% vs 31.1%, aRR 0.76 [95% CI 0.58, 1.00], *p* = 0.047). The risk was likewise decreased for sustained weight gain > 1.34 SD in the first 2 years of life (7.7% vs 17.1%, aRR 0.55 [95% CI 0.34, 0.88], *p* = 0.014). From five weight gain trajectories identified, there were more intervention offspring in the “normal” weight gain trajectory characterised by stable weight SDS around 0 SD from birth to 2 years (38.8% vs 30.1%, RR 1.29 [95% CI 1.03, 1.62], *p* = 0.029).

**Conclusions:**

Supplementation with *myo*-inositol, probiotics, and additional micronutrients preconception and in pregnancy reduced the incidence of rapid weight gain and obesity at 2 years among offspring. Previous reports suggest these effects will likely translate to health benefits, but longer-term follow-up is needed to evaluate this.

**Trial registration:**

ClinicalTrials.gov, NCT02509988 (Universal Trial Number U1111-1171–8056). Registered on 16 July 2015.

**Supplementary Information:**

The online version contains supplementary material available at 10.1186/s12916-024-03246-w.

## Background

The first 1000 days from conception to 2 years is a critical window for influencing later growth and body composition, including the future risks of underweight and obesity [1, 2]. Increasing evidence also implicates preconception influences on adverse offspring health outcomes [3], leading to calls for new initiatives to improve preconception health and care [4]. While increasing observational data implicate important roles for maternal obesity, glycaemia, and micronutrient status before and during pregnancy in increasing the risk of child obesity [5–14], there are few randomised trials of preconception and pregnancy interventions that examine outcomes in early childhood [15, 16].

Several micronutrients have been related to offspring adiposity, for example, increased adiposity has been observed among offspring whose mothers were vitamin D deficient during pregnancy [8, 12, 14]. Likewise, B-vitamin deficiencies have been observed among mothers with diabetes [10, 11, 17, 18], which may contribute to increased offspring adiposity [9, 13]. Similarly, *myo*-inositol, a non-essential sugar alcohol involved in regulating glucose and lipid metabolism, has been postulated to counteract the effects of maternal dysglycaemia and dyslipidaemia on offspring adiposity [19], and probiotics have been proposed as beneficial in preventing gestational dysglycemia [20].

The rate of offspring postnatal weight gain may mediate associations between maternal micronutrient deficiencies and later obesity [8, 12]. Though there are several criteria used to define rapid infant weight gain, it is most frequently defined as an increase in weight of 0.67 standard deviations (SD) or more, which is equivalent to the upward crossing of one or more major percentile lines on a growth chart [21]. Other studies have used posteriori methods to describe patterns of infant weight gain [22, 23]. Regardless of the definition used, rapid weight gain in infancy has consistently been associated with increased cardiometabolic risk [22–26].

In this context, the Nutritional Intervention Preconception and During Pregnancy to Maintain Healthy Glucose Levels and Offspring Health (NiPPeR) study provides an opportunity to examine the impact of maternal preconception and antenatal nutritional supplementation on offspring outcomes [27]. Women were recruited prior to pregnancy from three study centres (the UK, Singapore, and New Zealand) and were randomly allocated to receive a twice-daily nutritional beverage containing *myo*-inositol, probiotics, and additional micronutrients or a control beverage containing standard pregnancy micronutrients. The primary outcome of the trial was maternal glycaemia at 28 weeks of gestation; however, there were no differences between the intervention and control groups, including in the incidence of gestational diabetes [28]. Offspring postnatal weight gain and early childhood obesity were pre-specified secondary outcomes of the NiPPeR trial [27]. We aimed to determine whether preconception and antenatal supplementation with *myo*-inositol, probiotics, and additional micronutrients would optimise offspring body size and growth in the first two years of life.

## Methods

### Participants

Participants were offspring born to mothers participating in the NiPPeR study [27]. The detailed inclusion criteria of the NiPPeR study are described elsewhere [27]. Briefly, women were recruited between August 2015 and May 2017 and were eligible to participate if they were aged 18 to 38 years, were planning to conceive within 6 months, and had future maternity care planned at one of the study centres (Southampton, UK; Singapore; Auckland, New Zealand). Of the 1729 women randomised, 586 had births ≥ 24 weeks of gestation between April 2016 and January 2019 [28]. Of these births, six children were excluded from our analyses due to neonatal death (*n* = 1), stillbirth (*n* = 1), and congenital anomalies that may influence growth (*n* = 4), and four children were excluded due to missing data on key covariates (maternal BMI [*n* = 1] and maternal smoking during pregnancy [*n* = 3]).

### Ethics, consent, and permissions

The NiPPeR trial was registered on 16 July 2015 (ClinicalTrials.gov NCT02509988; Universal Trial Number U1111-1171–8056) and was conducted according to the guidelines laid down in the Declaration of Helsinki. Ethics approval was granted by the appropriate committees: Southampton – Health Research Authority National Research Ethics Service Committee South Central Research Ethics Committee (15/SC/0142); Singapore – the National Healthcare Group Domain Specific Review Board Singapore (2015/00205); and New Zealand – the Northern A Health and Disability Ethics Committee New Zealand (15/NTA/21). Written informed consent was obtained from the mothers of the included offspring.

### Nutritional intervention

The NiPPeR study was a randomised controlled trial with women allocated in a 1:1 ratio to either the control or intervention group with stratification by site and ethnicity. The trial was double-blinded for the primary outcome, with ongoing blinding of mothers throughout the study and follow-up. The NiPPeR study intervention was a twice-daily powdered drink supplement consumed preconception and throughout pregnancy. The control group were provided with a formulation with similar sensory characteristics. Both the intervention and control supplements contained folic acid (400 μg/day), iron (12 mg/day), calcium (150 mg/day), iodine (150 μg/day), and β-carotene (720 μg/day). The intervention additionally contained *myo*-inositol (4 g/day), vitamin D (10 μg/day), riboflavin (1.8 mg/day), vitamin B6 (2.6 mg/day), vitamin B12 (5.2 μg/day), zinc (10 mg/day), and probiotics (*Lactobacillus rhamnosus* NCC 4007 [CGMCC 1.3724] and *Bifidobacterium animalis* species lactis NCC 2818 [CNCM I-3446]).

### Anthropometry

Anthropometric measurements were analysed at birth, 3 weeks, 6 weeks, 3 months, 6 months, 1 year, and 2 years. Birthweight was obtained from hospital records. Subsequent weights in infancy were measured naked to the nearest 1 g using SECA 376 scales (SECA, Hamburg, Germany) by the research teams. At 2 years, weight was measured in a dry diaper or underwear to the nearest 100 g using SECA 899 scales. Recumbent crown-heel length was measured to the nearest 0.1 cm using a neonatometer (Holtain Ltd., Crymych, UK) or infantometer (Holtain Ltd.). At 2 years, standing height was measured to the nearest 0.1 cm using a SECA 213 portable stadiometer. Weight, length, or height (henceforth referred to as length SDS) and BMI SDS were calculated using the WHO Child Growth Standards adjusted for age and sex [29].

Data were subsequently screened for outlying measurements (> 4 or <  − 4 SD) and those where there was > 2 SD change between consecutive visits. These measurements were plotted using the WHO Child Growth Standards, and observations were omitted if not in keeping with the child’s growth trajectory from multiple measurements over the first 2 years. If the first length measurement was obtained beyond day 3, but prior to or on day 10, length was adjusted according to WHO age- and sex-specific length velocities [30]. No other length measurements at any other time points were adjusted. Sensitivity analyses were run excluding adjusted birth lengths, and the results were unchanged. Subsequently, results are reported for the primary analyses only.

### Outcomes

The main outcome of the present analyses was BMI SDS at 2 years, specifically average group estimates and risk of obesity [31] (> 1.65 SD, i.e. > 95th percentile). As differences in BMI SDS may be attributable to weight and/or length, weight and length SDS at 2 years were also examined. Findings were confirmed by seeking consistency with additional outcomes including weight, length, and BMI over the first 2 years based on repeated measurements, changes in auxological parameters from birth (i.e. ∆ weight, length, and BMI SDS), rapid weight gain derived as detailed below, and a posteriori-derived weight gain trajectories.

### Data analysis

Data were analysed to assess the effects of the NiPPeR intervention (i.e. treatment vs control) on the main outcome of the present study (BMI SDS at 2 years). We also carried out sensitivity analyses for the main outcome, including only participants (97.1%) with good adherence to the trial protocol assessed by sachet counting (defined a priori as ≥ 60% of the sachets taken), which was confirmed by elevated plasma maternal 25-hydroxyvitamin D concentrations among mothers in the intervention group at the 28-week OGTT [28]. Results from sensitivity analyses conducted using the a priori definition of good adherence were comparable, so these are not additionally reported. Notably, approximately half of the cohort had high adherence > 90%; therefore, additional sensitivity analyses were conducted.

BMI, weight, and length SDS at 2 years were analysed using general linear models that included an indicator variable for randomisation group, with adjustment for study site (a baseline randomisation factor; the UK/Singapore/New Zealand), baseline imbalances between randomisation groups [parity (nulliparous/multiparous) and maternal smoking during pregnancy (none/passive or active)], factors strongly associated with offspring growth [gestational age at birth (weeks) and maternal pre-pregnancy BMI (kg/m^2^) or height (cm) depending on the outcome], and infant sex (male/female) to enable evaluation of any sex-specific effects of the intervention. Notably, we did not adjust for birthweight in any analyses as the intervention was taken prior to and during pregnancy. Therefore, birthweight may be, in part, determined by the intervention [3, 15]. Two-year BMI SDS data were also analysed using logistic regression to determine if the distribution of those with obesity was similar between the intervention and control groups.

Additional analyses were conducted to examine the potential differences in BMI, weight, and length SDS trajectories in the first 2 years of life. First, data were modelled using linear spline linear mixed-effects models [32]. Data for BMI, weight, and length SDS from birth to 2 years were fitted using the lme4 package (v1.1–25) in R (v4.0.3, R Foundation for Statistical Computing, Vienna, Austria). Knots were placed at the quantiles of the age distribution, and models with two to four knots were compared. Nonlinear individual trajectories were allowed by including a random effects spline with one knot at the median. The best fitting models were selected according to the lowest Bayesian information criterion [10].

In addition, BMI, weight, and length SDS from birth to 2 years were analysed using adjusted linear mixed models with a repeated measures design including visit and a visit × randomisation group interaction term to enable group comparisons at each time point to be assessed. These analyses were restricted to include only measurements obtained within given visit windows: birth (0 to 3 days), 3 weeks (16 to 26 days), 6 weeks (37 to 54 days), 3 months (81 to 108 days), 6 months (169 to 204 days), 1 year (351 to 386 days), and 2 years (700 to 760 days).

The changes (Δ) in BMI, weight, and length SDS from birth to 2 years were analysed using general linear models, adjusted using the parameters mentioned above. Analyses were initially restricted to include only offspring data from visits where both length and weight data were available. However, as the above analyses primarily attributed differences in BMI SDS to weight, further analyses were conducted based on weight SDS only, for which there were more complete data, with no imputation of missing data. We analysed the risk of rapid weight gain, defined a priori as an increase in weight SDS greater than 0.67 SD [21]. Rapid weight gain (> 0.67 SD) from birth to 1 year has previously been found to be more strongly associated with later obesity than the same SD gain observed over the first 2 years of life [21]. This is not surprising, as the arbitrary 0.67 SD criterion over 1 year implies a higher velocity than when considered over 2 years. As such, we also examined rapid weight gain from birth to 2 years, defined as an increase greater than 1.34 SD, equivalent to upwards crossing of two or more major percentile lines, which represents sustained weight gain over the first 2 years.

Finally, exploratory analyses were conducted to identify a posteriori weight gain trajectories within our cohort by latent class growth analysis (LCGA) using the lcmm package (v2.0.0) in R and the previously described methods for linear trajectory modelling. Weight SDS and exact age at measurement were included in the analysis. The optimal number of distinct and interpretable classes was chosen according to Bayesian information criterion, log-likelihood, the median posterior probability of assignment of at least 70%, and class assignment of at least 5% [32]. The unadjusted risk ratios of class assignments were then determined using logistic regression, with class assignments coded into dummy variables. Sensitivity analyses were run including only offspring with three or more measurements in the first 2 years of life. Details of trajectory modelling, including sensitivity analyses, can be found in Additional file 1.

Descriptive statistics are reported as means ± SD or *n* (%), with differences between the groups evaluated using the chi-square tests and independent samples *t*-tests. Data for continuous outcomes are reported as least squares means (adjusted means) with respective 95% confidence intervals (CI), and the effect sizes are reported as adjusted mean differences (aMD) and 95% CI when comparing the groups. Data for binary outcomes are reported as the adjusted risk ratios (aRR) and 95% CI. All tests were two-tailed and carried out using SAS version 9.4 (SAS Institute, Cary, NC, USA) or R version 4.0.3. The statistical significance level was two-tailed and set at *p* < 0.05.

## Results

### Participant flow chart

**Fig. 1:**
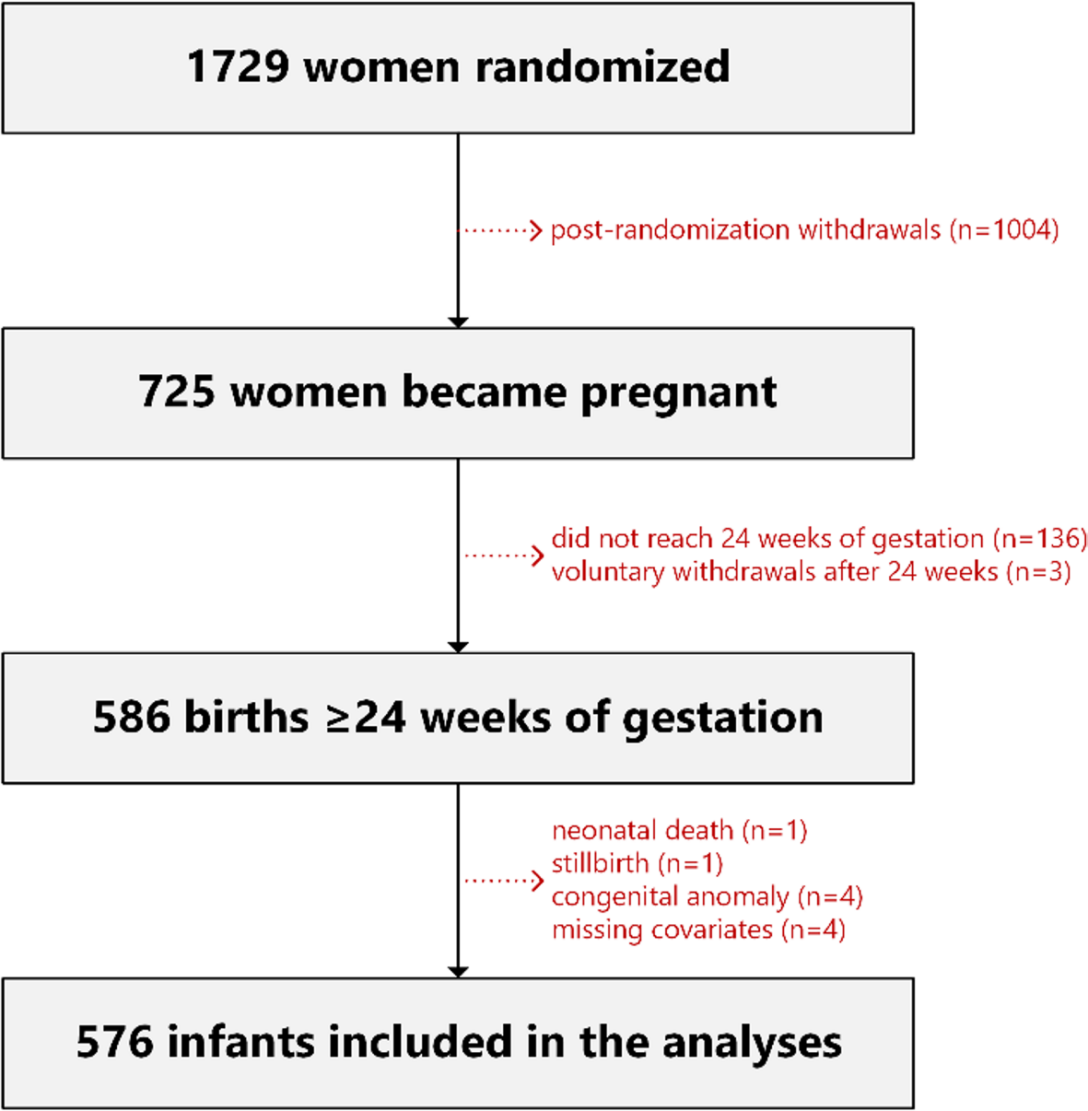
CONSORT flow diagram

Anthropometric data from the first 2 years of life were available from 576 offspring (Fig. 1). Additional file 2: Table S1 details the number of measurements available at each time point.

### Characteristics of the study population

Characteristics of the study population included in this study are outlined in Table 1. Most babies were born at term (91.8%) and had birthweights that were appropriate for gestational age (85.1%). The mean maternal BMI and other baseline characteristics were similar in the two randomisation groups, except fewer mothers in the intervention group were nulliparous, and intervention offspring were less likely to be exposed to passive smoking in utero.

**Table 1:** Characteristics of the study population

	Intervention (*n* = 287)	Control (*n* = 289)
Study site		
UK	95 (33.1%)	92 (31.8%)
Singapore	83 (28.9%)	82 (28.4%)
New Zealand	109 (38.0%)	115 (39.8%)
Maternal ethnicity^a^		
White Caucasian	174 (60.6%)	167 (57.8%)
Chinese	70 (24.4%)	73 (25.3%)
South Asian	16 (5.6%)	15 (5.2%)
Malay	11 (3.8%)	12 (4.2%)
Others	16 (5.6%)	22 (7.6%)
Maternal BMI (kg/m^2^)	24.5 ± 5.1	25.1 ± 5.8
Maternal height (cm)	164.6 ± 6.6	163.7 ± 7.0
Parity^b^		
Nulliparous	166 (57.8%)	201 (69.6%)
Multiparous	121 (42.2%)	88 (30.4%)
Maternal smoking during pregnancy		
None	256 (89.2%)	234 (81.0%)
Passive smoking	22 (7.7%)	45 (15.6%)
Active smoking	9 (3.1%)	10 (3.4%)
Household income quintile		
5 (lowest)	2 (0.4%)	5 (0.9%)
4	21 (3.7%)	21 (3.7%)
3	54 (9.4%)	68 (11.8%)
2	107 (18.6%)	95 (16.5%)
1 (highest)	92 (16.0%)	90 (15.6%)
Not available	11 (1.9%)	10 (1.7%)
Infant sex		
Male	138 (48.1%)	130 (45.0%)
Female	149 (51.9%)	159 (55.0%)
Gestational age (weeks)^c^	39.3 ± 1.5	39.2 ± 1.7
Preterm	15 (5.2%)	27 (9.3%)
Term	269 (93.7%)	260 (90.3%)
Post-term	3 (1.0%)	2 (0.7%)
Birthweight (g)	3354 ± 524	3300 ± 542
Birthweight SDS^d^	0.00 ± 0.92	− 0.04 ± 0.93
SGA	22 (7.7%)	21 (7.3%)
AGA	244 (85.0%)	246 (85.1%)
LGA	21 (7.3%)	22 (7.6%)
Any breastfeeding		
Yes	273 (96.5%)	275 (98.6%)
No	10 (3.5%)	4 (1.4%)
Missing	4	10
Never exclusively breastfed^e^		
Yes	162 (57.5%)	150 (54.4%)
No	120 (42.6%)	126 (45.7%)
Missing	5	13
Exclusive breastfeeding duration (weeks)^e,f^	7.7 ± 9.8	7.1 ± 9.7
Missing	5	13
Any breastfeeding duration (weeks)^f^	38.6 ± 18.2	36.1 ± 19.9
Missing	5	10

*Abbreviations*: *AGA* Appropriate-for-gestational-age, *BMI* Body mass index, *LGA* Large-for-gestational-age, *SDS* Standard deviation score, *SGA* Small-for-gestational-age

Data are mean ± SD or *n* (%)

^a^South Asian includes Indian, Pakistani, and Bangladeshi mothers. “Others” includes mothers of mixed, Black, or Polynesian ethnicity

^b^Multiparous includes mothers with one or more births > 24 weeks of gestation

^c^Preterm defined as birth prior to 37^0/7^ weeks of completed gestation, term as birth between 37^0/7^ and 41^6/7^ weeks of completed gestation, and post-term as birth at or beyond 42^0/7^ weeks of completed gestation

^d^Calculated using the UK–WHO reference [33]; SGA defined as below the 10th percentile (− 1.282 SD) and LGA as above the 90th percentile (1.282 SD)

^e^Exclusive breastfeeding defined as the infant having never received any water, formula, or other liquid or solid food, except for oral rehydration solution or drops/syrups of vitamins, minerals, or medicines

^f^Those who were never breastfed or never exclusively breastfed were assigned a value of 0

### BMI, weight, and length at 2 years

Data on both weight and length were available from 484 offspring at 2 years. Maternal characteristics of those who provided data at 2 years were largely comparable to those without data (Additional file 2: Table S2), except a greater proportion were from New Zealand (42.8% vs 18.5%, *p* < 0.001), were of Chinese ethnicity (26.9% vs 14.1%, *p* = 0.006), and were nulliparous (65.5% vs 54.3%, *p* = 0.042). Furthermore, included offspring had moderately greater gestational ages at birth (39.3 ± 1.5 weeks vs 38.9 ± 2.2 weeks, *p* = 0.011) (Table S2).

BMI SDS tended to be lower among offspring of mothers in the intervention group at 2 years (aMD − 0.14 SD [95% CI − 0.30, 0.02], *p* = 0.09) (Table 2). Fewer intervention offspring had obesity (> 1.65 SD; 95th percentile) at 2 years (*n* = 22 [9.2%] vs *n* = 44 [18.0%], *p* = 0.005). Adjusting for study site, infant sex, parity, maternal smoking, maternal pre-pregnancy BMI, and gestational age at birth, intervention offspring had approximately half the risk of having obesity at 2 years compared to controls (Arr 0.51 [95% CI 0.31, 0.82], *p* = 0.006). The results were similar following adjustment for exclusive and any breastfeeding duration (Additional file 2: Table S3).

**Table 2:** Body mass index (BMI), weight, and length standard deviation scores (SDS) at 2 years among offspring, according to the randomisation group (*n*=484)

	Intervention, *n* = 239	Control, *n* = 245	aMD	*p*
BMI SDS	0.53 (0.38, 0.67)	0.67 (0.53, 0.81)	− 0.14 (− 0.30, 0.02)	0.09
Weight SDS	0.17 (0.03, 0.32)	0.29 (0.15, 0.43)	− 0.12 (− 0.28, 0.04)	0.15
Length SDS	− 0.31 (− 0.46, − 0.16)	− 0.20 (− 0.35, − 0.05)	− 0.11 (− 0.28, 0.06)	0.19

*Abbreviations*: *aMD* Adjusted mean difference, *SDS* Standard deviation scores

Data are least squares means (i.e. adjusted means) and respective 95% confidence intervals from general linear models adjusted for study site (the UK/Singapore/New Zealand), infant sex (male/female), parity (nulliparous/multiparous), maternal smoking (none/active or passive), maternal pre-pregnancy BMI (for BMI SDS and weight SDS) or maternal height (for length SDS), and gestational age at birth

Sensitivity analyses run including only children born to mothers with adherence > 90% (*n* = 245) showed a greater reduction in average BMI (− 0.21 SD [95% CI − 0.44, 0.02], *p* = 0.07) and a 63% reduction in the risk of obesity at 2 years (*n* = 9 [7.1%] vs *n* = 21 [17.8%]; aRR 0.37 [95% CI 0.17, 0.80], *p* = 0.012).

### BMI, weight, and length in the first 2 years of life

**Fig. 2:**
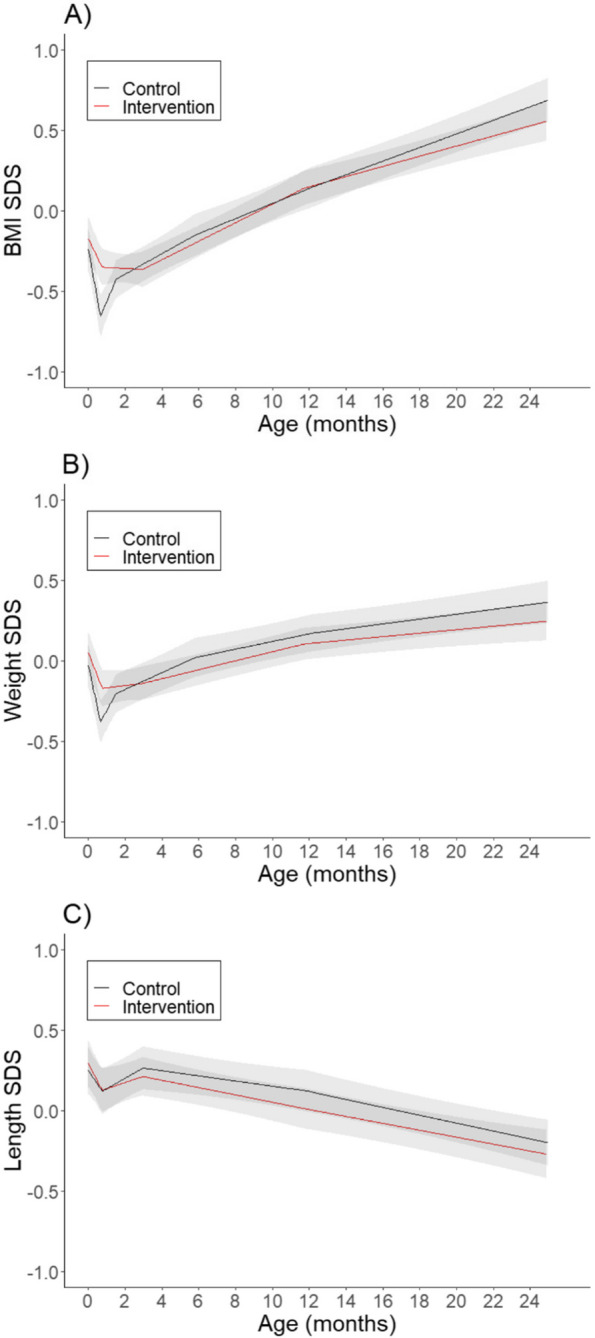
Mean **A** body mass index (BMI), **B** weight, and **C** length standard deviation score (SDS) trajectories from linear spline linear-mixed effects models. The estimated mean trajectories in intervention (red) and control (black) offspring. Shaded areas around the mean trajectories represent 95% confidence intervals

The predicted BMI, weight, and length SDS trajectories from birth to 2 years are depicted in Fig. 2. From birth, there was an initial period of BMI SDS decrease in both groups, followed by a plateau to 3 months among intervention offspring, but a period of rapid BMI gain to 6 weeks among control offspring (Fig. 2). BMI SDS then increased modestly through to 2 years, particularly in the control group (Fig. 2). The initial period of BMI SDS decrease was largely attributable to a reduction in weight SDS, with the slope of the first segment (from birth to approximately 3 weeks) being almost 50% greater among control offspring (Fig. 2).

**Fig. 3:**
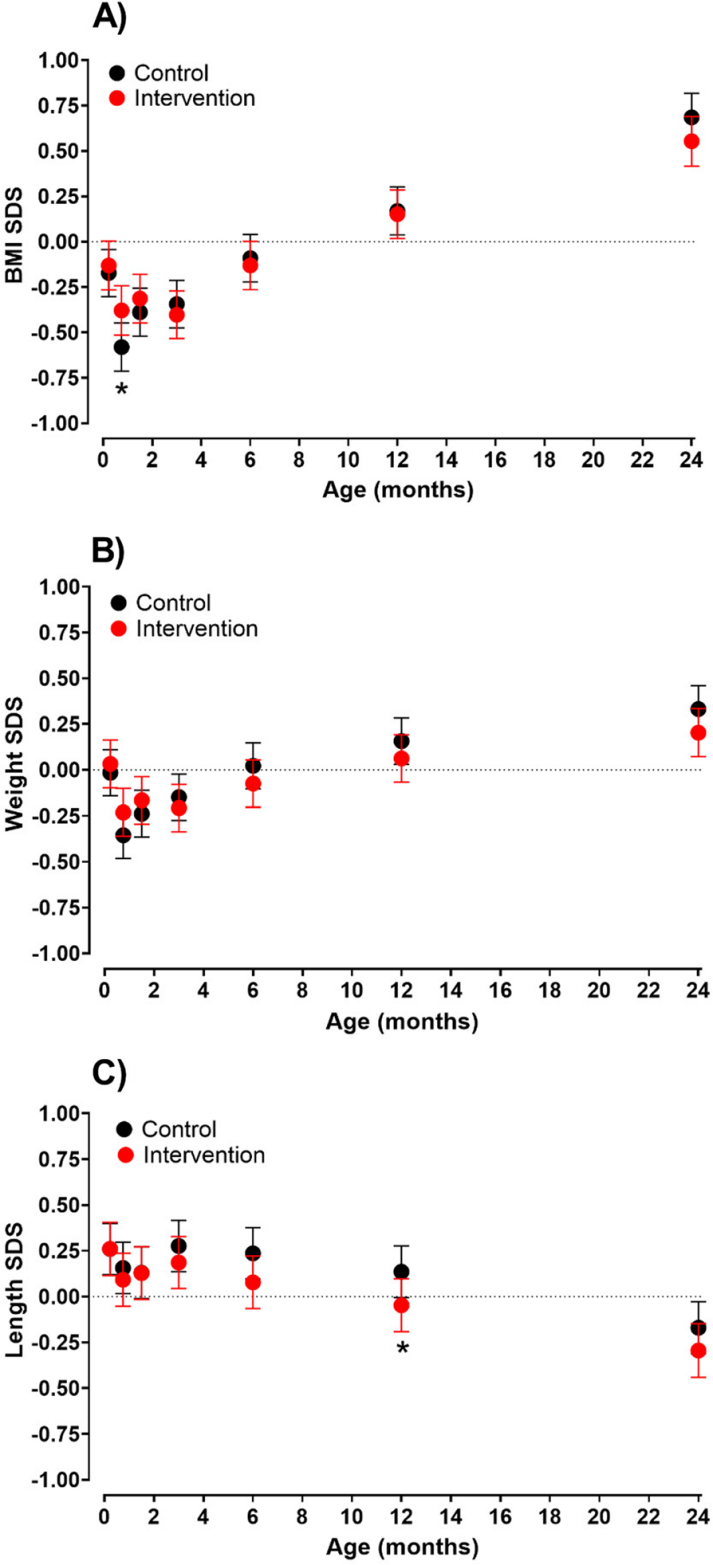
Least squares means (adjusted means) and 95% confidence intervals of **A** body mass index (BMI), **B** weight, and **C** length standard deviation scores (SDS) by visit from repeated measures linear mixed models (*n* = 563) for intervention (red) and control (black) offspring. *p < 0.05

In adjusted repeated measures analyses, the mean BMI SDS did not differ between the control and intervention groups, except at the 3-week visit, where intervention offspring had a shallower drop in BMI SDS (aMD + 0.20 SD [95% CI 0.03, 0.37], *p* = 0.021) (Fig. 3). The results were similar when the analyses were confined to offspring with measurements at all visits (*n* = 201) (Additional file 2: Fig. S1).

### Change in BMI, weight, and length

Analyses of changes in auxological parameters (∆ analyses) showed an increase in BMI SDS from birth to 2 years among both randomisation groups. The increase in BMI SDS was lower among intervention offspring (aMD − 0.30 SD [95% CI − 0.51, − 0.09], *p* = 0.006), with this difference being driven by greater ∆ weight SDS among control offspring (aMD 0.27 SD [95% CI 0.10, 0.44], *p* = 0.002) without a similar proportional gain in length SDS (aMD SD 0.14 [95% CI − 0.06, 0.35], *p* = 0.17). When delta analyses were re-run including only children born to mothers with high adherence (*n* = 220), the effect size increases to − 0.44 SD ([95% CI − 0.72, − 0.16], *p* = 0.002) for BMI SDS and − 0.39 SD ([95% CI − 0.62, − 0.17], *p* < 0.001) for weight SDS.

Although the intervention effect was comparable across sites, mean ∆ BMI SDS to 2 years was higher among Singaporean offspring in both the intervention and control groups (overall—SG, 1.07 ± 1.07; the UK, 0.28 ± 1.15; NZ, 0.72 ± 1.15, *p* < 0.001). Group differences were comparable when preterm offspring (*n* = 42) were removed from the analyses (data not shown), though preterm offspring experienced greater increases in BMI SDS than term offspring (overall—1.92 ± 1.09 vs 0.67 ± 1.12, *p* < 0.001).

### Rapid weight gain

After adjustment for confounding factors, intervention offspring had between 24 and 45% lower risk of rapid weight gain depending on the threshold and time period considered (Table 3). The greatest difference was observed for sustained rapid weight gain > 1.34 SD in the first 2 years (Table 3). Singaporean offspring had greater risks of weight gain > 0.67 SD from birth to 1 year compared to the UK and New Zealand offspring (63 [41.7%] vs 75 [20.2%], aRR 1.81 [95% CI 1.36, 2.39], *p* < 0.001), though there were no site differences in the risk of weight gain > 1.34 SD from birth to 2 years.

**Table 3:** Adjusted risk ratios and 95% confidence intervals of rapid weight gain from birth to 1 or 2 years

	*n* (%)		aRR (95% CI)	*p*
	Intervention	Control		
> 0.67 SD from birth to 1 year	58 (21.9%)	80 (31.1%)	0.76 (0.58, 1.00)	**0.047**
> 1.34 SD from birth to 2 years	19 (7.7%)	43 (17.1%)	0.55 (0.34, 0.88)	**0.014**

Data are adjusted risk ratios and respective 95% confidence intervals from logistic regression adjusted for study site (the UK/Singapore/New Zealand), infant sex (male/female), parity (nulliparous/multiparous), maternal smoking (none/active or passive), maternal pre-pregnancy BMI, and gestational age at birth. Statistically significant comparisons (*p* < 0.05) are shown in bold

Increasing gestational age was associated with a lower risk of rapid weight gain; for example, each weekly increase in gestational age was associated with a 37% reduction in the risk of rapid weight gain > 1.34 SD from birth to 2 years (aRR 0.63 [95% CI 0.57, 0.68], *p* < 0.001), whereas preterm birth was associated with an approximately sixfold increase in risk (aRR 5.93 [95% CI 3.87, 9.10], *p* < 0.001) compared with term births. Approximately 45% of offspring who experienced rapid weight gain > 1.34 SD from birth to 2 years were preterm or born small for gestational age. However, the proportion of such cases was similar in the intervention and control groups (8 [42.1%] vs 20 [46.5%], *p* = 0.75). When the analysis was re-run excluding these offspring (*n* = 28), a reduced risk of rapid weight gain > 1.34 SD from birth to 2 years with the intervention remained (11 [5.1%] vs 23 [10.9%], aRR 0.47 [95% CI 0.24, 0.93], *p* = 0.030).

### Latent class growth analysis

**Fig. 4:**
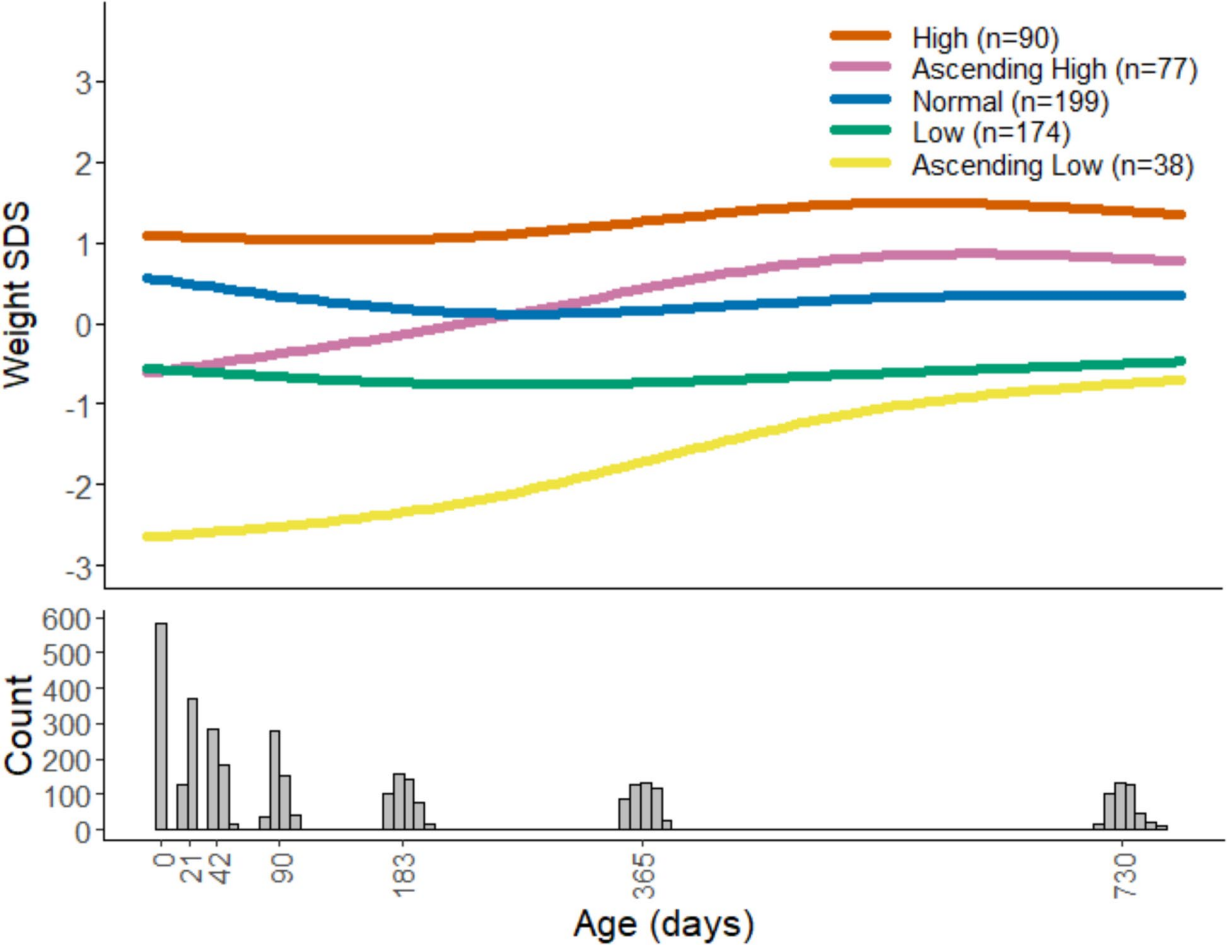
Predicted latent class growth analysis weight standard deviation score (SDS) trajectories

Five distinct trajectories were identified using LCGA. These included weight gain trajectories characterised by weight SDS which were low, average, and high from birth (“low”, “normal”, and “high”, respectively) and those characterised by high weight gain either from very low weight SDS at birth to low weight SDS at 2 years or from low weight SDS at birth to moderate weight SDS at 2 years (“ascending low” and “ascending high”, respectively) (Fig. 4).

Intervention offspring were 30% more likely to be assigned to the normal trajectory (112 [38.8%] vs 87 [30.1%], RR 1.29 [95% CI 1.03, 1.62], *p* = 0.029) and 48% less likely to be assigned to the ascending low weight gain trajectory (13 [4.5%] vs 25 [8.7%], RR 0.52 [95% CI 0.27, 1.00], *p* = 0.046). There was also a trend towards fewer intervention offspring being assigned to the high trajectory (37 [12.8%] vs 53 [18.3%], RR 0.69 [95% CI 0.47, 1.02], *p* = 0.063). Singaporean offspring were less likely to be assigned to the normal class than the UK and New Zealand offspring (30 [18.1%] vs 169 [41.0%], *p* < 0.001) but were more likely to be assigned to the two ascending classes (51 [30.7%] vs 64 [15.5%], *p* < 0.001).

## Discussion

We have demonstrated that maternal preconception and antenatal supplementation with *myo*-inositol, probiotics, and additional micronutrients were associated with a lower risk of rapid infant weight gain and obesity among offspring at 2 years. A posteriori trajectory modelling supported these findings, with more offspring in the intervention group assigned to a normal weight gain trajectory. As previous research has associated rapid infant weight gain with later obesity [21, 24, 34, 35], these findings suggest a protective effect of preconception and antenatal nutritional supplementation, which may have long-term benefits to the offspring. For example, high childhood BMI has been associated with an increased risk of cardiovascular disease and subsequent obesity in adulthood [36–38], which is consistent with reported findings for a tendency for obesity to track from childhood [24, 37].

*Myo*-inositol supplementation from early gestation has previously been associated with reductions in the incidence of gestational diabetes, preterm birth, and excessive foetal growth [39–41]. In the NiPPeR study, despite no differences in maternal glycaemia and incidence of gestational diabetes, there was a reduction in late preterm birth [28]. In the current study, results of offspring auxology were unchanged following adjustment for gestational age at birth and after exclusion of preterm births. Thus, the observed lower risk of rapid infant weight gain appears to be independent of the protective effects of the intervention on preterm birth [28]. Furthermore, as there were no intervention effects on gestational glycaemia, our findings also cannot be attributed to improvements in maternal glycaemic regulation [28].

In contrast to previous studies of antenatal *myo*-inositol supplementation, the NiPPeR maternal population was a self-selected cross-section from the community rather than a selected high metabolic risk group, and there were differences in the timing of commencement (i.e. preconception vs early pregnancy) and formulation (i.e. single- vs multi-nutrient) of the intervention. Nonetheless, the risk for rapid weight gain and obesity at 2 years was similarly reduced among metabolic risk groups (e.g. maternal overweight/obesity and gestational diabetes) (Additional file 2: Table S4). To our knowledge, no study of antenatal *myo*-inositol supplementation has assessed offspring growth, and the role of inositols in foetal growth and fat accretion remains poorly understood. However, it has been postulated that inositol is involved in the cross-talk across the maternal-placental-foetal axis to regulate foetal growth and development [19].

Other key micronutrients in the intervention have been associated with offspring adiposity. Low vitamin D status in late pregnancy has been associated with lower fat mass in offspring at birth, but subsequent elevated fat mass at 6 years, which is suggestive of rapid postnatal weight gain [12]. Similarly, in a study of over 60,000 Norwegian children, increasing maternal vitamin D intake was associated with lower weight gain trajectories and reduced odds of rapid weight gain and childhood overweight [8]. Pre-pregnancy maternal BMI was a modulating factor, and contrasting trends were observed among children of mothers with pre-pregnancy overweight compared with pre-pregnancy normal weight [8]. Furthermore, there is evidence to suggest a plateauing of the effect, where serum vitamin D was inversely associated with offspring adiposity, plateauing above serum levels of approximately 64 nmol/L [12]. While serum levels of vitamin D were increased among intervention mothers in the NiPPeR trial (manuscript in preparation), a previous study [42] found that supplementation with vitamin D from mid-pregnancy, when combined with calcium, iron, and folic acid (vs control supplement without vitamin D), did not reduce the incidence of gestational diabetes, preterm birth, or offspring anthropometry at birth or 1 year. The population studied had a high prevalence of vitamin D insufficiency, but the lack of an observed difference might have been related to the multiple-micronutrient format or the timing of the intervention [42]. B-group vitamins have also been related with maternal glycaemia [11, 17, 18, 43], with maternal vitamin B12 deficiency associated with increased insulin resistance and adiposity among offspring [9, 13, 44]. Thus, the beneficial effects of supplementation on infant weight gain among our cohort could be related to improvements in nutritional sufficiency, though the mechanisms are unclear.

In our cohort, rapid weight gain was relatively common, with 26% of infants experiencing rapid weight gain (> 0.67 SD) in the first year of life. Previously, a meta-analysis of 17 studies reported incidences of rapid infant weight gain in the range of 12 to 54%. Rapid weight gain was associated with 3.7 times increased odds of later overweight or obesity, though there was substantial heterogeneity in the time period examined and the age at outcome assessment [21]. Similar associations have been found when analysing a posteriori weight gain trajectories, with classes characterised by rapid weight gain having the highest risk of increased adiposity and cardiometabolic perturbations later in life [22–26].

The weight gain trajectories between birth and 2 years identified in our cohort are similar to those previously described in contemporary paediatric cohorts [22, 23, 25]. In The Applied Research Group for Kids (TARget Kids!) study, the “Rapid Accelerating” class (characterised by increasing BMI SDS from 6 months) was associated with increased age- and sex-standardised cardiometabolic risk scores at 3 to 5 years, but few children (1%) were assigned to this class [22]. Similarly, among children in the Growing Up in Singapore Towards healthy Outcomes (GUSTO) study, four BMI SDS trajectories were identified in the first 2 years of life—“stable low”, “normal”, “stable high”, and “rapid BMI SDS gain after 3 months”—equivalent to our low, normal, high, and accelerating high classes. The stable high and rapid BMI SDS gain groups were associated with increased obesity at 5 years [23]. More recently, trajectories have been developed among the GUSTO cohort incorporating data from birth to 6 years [25]. Five BMI SDS trajectories were identified, with three stable trajectories (equivalent to the trajectories previously described), as well as two accelerating trajectories, with elevated foetal abdominal circumference and BMI acceleration immediately after birth or normal foetal growth with BMI acceleration after infancy. These accelerating trajectories were associated with increased abdominal fat, liver fat, insulin resistance, and hypertension at 6 years [25]. In GUSTO, ethnic differences were apparent between growth trajectories, with Malay and Indian offspring being more likely to be in the accelerating BMI gain trajectories [23]. We similarly observed increased assignment to the ascending classes among Singaporean offspring, though the NiPPeR study was not powered to explore ethnic differences and thus could not determine if patterns differed between Chinese, South Asian, and Malay Singaporean offspring. Together, these findings suggest that offspring in our trial assigned to the two ascending classes may be at an increased risk of later cardiometabolic perturbations, and this may differ by ethnicity.

There are several strengths to this study. The NiPPeR study is a multinational, randomised controlled trial with extensive ongoing prospective data collection commencing from the preconception period. Anthropometric data were collected frequently in the first 2 years starting from birth, reducing the risk of spurious findings. Cross-sectional and longitudinal data were considered in the analyses, including multiple approaches for modelling nonlinear growth trajectories [25, 32]. Although each statistical method has its limitations, for example, LCGA requires subjective decisions about the number and placement of knots and the optimal number of classes to be retained [32], results were consistent across the statistical methods employed.

Limitations to the current study include that anthropometric measurements cannot distinguish between fat- and fat-free masses; therefore, it is unclear if the observed differences in weight gain are attributable to increased fat mass deposition or higher fat-free mass. Nonetheless, a study among Ethiopian infants attributed catch-up weight gain to fat mass [45], with higher fat mass accretion in infancy associated with increased adiposity at 5 years [46]. Further work is required to establish if the weight gain trajectories observed in the NiPPeR cohort are associated with fat or fat-free masses and if the intervention effects on early postnatal weight gain are associated with reduced adiposity later in life, as well as independent validation of the findings. Furthermore, the intervention’s multinutrient formulation limits the ability to determine the effects of specific nutrients and, thus, potential mechanisms.

## Conclusions

In conclusion, our analyses among NiPPeR trial offspring showed that preconception and antenatal supplementation with *myo*-inositol, probiotics, and additional micronutrients was associated with a lower risk of obesity at 2 years, which may be related to less rapid weight gain in infancy. Previous reports suggest these effects will likely translate to health benefits but longer-term follow-up is needed to evaluate this.

### Supplementary Information

**Additional file 1:** Details trajectory modelling methods, including sensitivity analyses. **Table S1.** Latent class growth analysis model summary statistics for weight standard deviation scores (SDS) from birth to 2 years. **Table S2.** Latent class growth analysis model summary statistics for weight standard deviation scores (SDS) from birth to 2 years including only offspring with ≥3 visits. **Table S3.** Risk ratios and 95% confidence intervals for the weight standard deviation scores (SDS) trajectories from the sensitivity analysis five class latent class growth analysis model. **Fig. S1.** Trajectories for latent class growth analysis six class model of weight standard deviation scores (SDS) from birth to 2 years. **Fig. S2.** Trajectories for latent class growth analysis five class model of weight standard deviation scores (SDS) from birth to 2 years. **Fig. S3.** Individual trajectories for latent class growth analysis six class model of weight standard deviation scores (SDS) from birth to 2 years. **Fig. S4.** Individual trajectories for latent class growth analysis five class model of weight standard deviation scores (SDS) from birth to 2 years. **Fig. S5.** Sensitivity analysis trajectories for latent class growth analysis six class model for weight standard deviation scores (SDS) from birth to 2 years including only offspring with ≥3 visits. **Fig. S6.** Sensitivity analysis trajectories for latent class growth analysis five class model for weight standard deviation scores (SDS) from birth to 2 years including only offspring with ≥3 visits. **Fig. S7.** Sensitivity analysis individual trajectories for latent class growth analysis six class model for weight standard deviation scores (SDS) from birth to 2 years including only offspring with ≥3 visits. **Fig. S8.** Sensitivity analysis individual trajectories for latent class growth analysis five class model for weight standard deviation scores (SDS) from birth to 2 years including only offspring with ≥3 visits.

**Additional file 2:** Additional cohort descriptive statistics and results from sensitivity analyses. **Table S1.** Number of anthropometric measurements at each visit by randomisation group. **Table S2.** Characteristics of the study population who provided data at 2 years. **Table S3.** Body mass index (BMI), weight, and length standard deviation scores (SDS) at 2 years among offspring, according to randomisation group (*n *= 481), with adjustment for breastfeeding duration. **Table S4.** Adjusted risk ratios and 95% confidence intervals of body mass index (BMI) at 2 years and rapid weight gain from birth to 1 or 2 years among sub-groups of different maternal metabolic risk. **Fig. S1.** Least squares means of A) body mass index (BMI), B) weight, and C) length standard deviation scores (SDS) by visit from repeated measures linear mixed models among offspring with measurements at each visit (*n *= 201). Figure shows the adjusted means and 95% confidence intervals for intervention (red) and control (black) offspring. *p <0.05.

## Data Availability

The datasets generated and/or analysed during the current study are not publicly available due to the participants not consenting to open access data sharing and this being an ongoing longitudinal study in which there will be further future analyses conducted but are available from the corresponding author upon reasonable request.

## Abbreviations

Amd: Adjusted mean difference
aRR: Adjusted risk ratio
BIC: Bayes information criteria
BMI: Body mass index
CI: Confidence interval
LCGA: Latent class growth analysis
NiPPeR: Nutritional Intervention Preconception and during Pregnancy to maintain healthy glucose levels and offspring health
SD: Standard deviation
SDS: Standard deviation score
WHO: World Health Organization
