# Supplementary material for: Impact of preconception and antenatal supplementation with myo-inositol, probiotics, and micronutrients on offspring BMI and weight gain over the first 2 years
Source: BMC Med. 2024 Jan 30;22:39. doi: 10.1186/s12916-024-03246-w (PMC10826220; doi:10.1186/s12916-024-03246-w)
Supplement: Supplementary file 1 — Additional file 1: Details trajectory modelling methods, including sensitivity analyses. Table S1. Latent class growth analysis model summary statistics for weight standard deviation scores (SDS) from birth to 2 years. Table S2. Latent class growth analysis model summary statistics for weight standard deviation scores (SDS) from birth to 2 years including only offspring with ≥3 visits. Table S3. Risk ratios and 95% confidence intervals for the weight standard deviation scores (SDS) trajectories from the sensitivity analysis five class latent class growth analysis model. Fig. S1. Trajectories for latent class growth analysis six class model of weight standard deviation scores (SDS) from birth to 2 years. Fig. S2. Trajectories for latent class growth analysis five class model of weight standard deviation scores (SDS) from birth to 2 years. Fig. S3. Individual trajectories for latent class growth analysis six class model of weight standard deviation scores (SDS) from birth to 2 years. Fig. S4. Individual trajectories for latent class growth analysis five class model of weight standard deviation scores (SDS) from birth to 2 years. Fig. S5. Sensitivity analysis trajectories for latent class growth analysis six class model for weight standard deviation scores (SDS) from birth to 2 years including only offspring with ≥3 visits. Fig. S6. Sensitivity analysis trajectories for latent class growth analysis five class model for weight standard deviation scores (SDS) from birth to 2 years including only offspring with ≥3 visits. Fig. S7. Sensitivity analysis individual trajectories for latent class growth analysis six class model for weight standard deviation scores (SDS) from birth to 2 years including only offspring with ≥3 visits. Fig. S8. Sensitivity analysis individual trajectories for latent class growth analysis five class model for weight standard deviation scores (SDS) from birth to 2 years including only offspring with ≥3 visits. [file 12916_2024_3246_MOESM1_ESM.docx]

**Impact of preconception and antenatal supplementation with *myo*-inositol, probiotics and micronutrients on offspring BMI and weight gain over the first two years**

Jaz Lyons-Reid^1^, José G. B. Derraik^1,2,3,4^, Timothy Kenealy^1,5^, Benjamin B. Albert^1^, J. Manuel Ramos Nieves^6^, Cathriona R. Monnard^6^, Phil Titcombe^7^, Heidi Nield^7^, Sheila J. Barton^7^, Sarah El-Heis^7,8^, Elizabeth Tham^9,10,11^, Keith M. Godfrey^7,8,#^, Shiao-Yng Chan^9,10,11,#^, Wayne S. Cutfield^1,12,#,*^ and the NiPPeR Study Group^‡^

^1^Liggins Institute, The University of Auckland, Auckland, New Zealand

^2^Department of Paediatrics: Child and Youth Health, Faculty of Medical and Health Sciences, The University of Auckland, Auckland, New Zealand

^3^Environmental-Occupational Health Sciences and Non-communicable Diseases Research Group, Research Institute for Health Sciences, Chiang Mai University, Chiang Mai, Thailand

^4^Department of Women's and Children's Health, Uppsala University, Uppsala, Sweden

^5^Department of Medicine and Department of General Practice and Primary Health Care, The University of Auckland, Auckland, New Zealand

^6^Nestlé Institute of Health Sciences, Nestlé Research, Société des Produits Nestlé S.A., Lausanne, Switzerland

^7^MRC Lifecourse Epidemiology Centre, University of Southampton, Southampton, United Kingdom

^8^NIHR Southampton Biomedical Research Centre, University of Southampton and University Hospital Southampton NHS Foundation Trust, Southampton, United Kingdom

^9^Singapore Institute for Clinical Sciences, Agency for Science, Technology and Research (A*STAR), Singapore

^10^Human Potential Translational Research Programme, Yong Loo Lin School of Medicine, National University of Singapore, Singapore

^11^Department of Obstetrics & Gynaecology, National University of Singapore, Singapore

^12^A Better Start – National Science Challenge, The University of Auckland, Auckland, New Zealand

Corresponding author: Wayne S. Cutfield, MD, Liggins Institute, University of Auckland, Private Bag 92019, Auckland, New Zealand. Email: w.cutfield@auckland.ac.nz. Ph: +64 9 923 4476

#These authors share joint last authorship.

‡Ryan Carvalho (ryan.carvalho@nestle.com), Julie Ann Castro (julie_castro@nuhs.edu.sg), Mary Cavanagh (m.cavanagh@auckland.ac.nz), Hsin Fang Chang (hsin_fang_chang@nuhs.edu.sg), Yap Seng Chong (obgcys@nus.edu.sg), Paula Costello (pc@mrc.soton.ac.uk), Vanessa Cox (vac@mrc.soton.ac.uk), Sevasti Galani (sevasti.galani@ucl.ac.uk), Judith Hammond (j.hammond@auckland.ac.nz), Nicholas C Harvey (nch@mrc.soton.ac.uk), Soo Min Han (clara.han@auckland.ac.nz), Mrunalini Jagtap (mrunalini.jagtap1@gmail.com), Chiara Nembrini (Chiara.Nembrini@rdls.nestle.com), Justin M O’Sullivan (justin.osullivan@auckland.ac.nz), Judith Ong (judith_ong@nuhs.edu.sg), Irma Silva-Zolezzi (irma.silvazolezzi@nestle.com), Wendy Sim (sin_nie_sim@nuhs.edu.sg), Vicky Tay (Vicky_tay@sics.a-star.edu.sg), Mya-Thway Tint (Mya_Thway_Tint@sics.a-star.edu.sg), Mark Vickers (m.vickers@auckland.ac.nz), Jui-Tsung Wong (csd3589@yahoo.com), Gladys Woon (gladys_woon@nuhs.edu.sg), Wen Lun Yuan (wenlun.yuan@inserm.fr)

Details on trajectory modelling

*A posteriori* weight gain trajectories were identified by latent class growth analysis (LCGA) using the hlme package in R. Age- and sex-specific weight standardised deviation scores (SDS) and exact ages at measurement were included in the analysis. Knots were placed at quantiles of the age distribution, with models run considering two, three, and four knots. The optimal number of distinct, interpretable classes was chosen according to Bayes information criteria (BIC), log likelihood, median posterior probability of assignment of at least 70%, and class assignment of at least 5%.

The inclusion of additional knots did not notably improve model fit; therefore, further analyses considered two-knot solutions. The six-class model had the lowest BIC and highest log likelihood of the models which met the minimum class size requirement (>5%) (**Table 1**). However, the five-class model was considered in subsequent analyses due to the similarity of the two “Normal” trajectories (i.e. “High to Normal” and “Low to Normal”) identified with the six-class model. Mean trajectories are shown in **Figures 1 and 2.** Individual trajectories according to class assignment are shown in **Figures 3 and 4**.

Sensitivity analyses were conducted including only offspring with three or more measurements in the first two years (**Table 2**). The trajectories identified were comparable to those identified in the main analyses (**Figures 5–8**). Results from logistic regression analyses were similar to the main findings (**Table 3**).

**Table 1 Latent class growth analysis model summary statistics for weight standard deviation scores (SDS) from birth to 2 years.**

|  | BIC | Log Likelihood | Entropy | Class 1 | Class 2 | Class 3 | Class 4 | Class 5 | Class 6 | Class 7 |
| --- | --- | --- | --- | --- | --- | --- | --- | --- | --- | --- |
| 2 knots | | | | | | | | | | |
| 1 | 9913.50 | -4940.83 | 1.000 | 100.00 |  |  |  |  |  |  |
| 2 | 8780.83 | -4358.58 | 0.812 | 60.31 | 39.69 |  |  |  |  |  |
| 3 | 8167.98 | -4036.24 | 0.861 | 51.89 | 7.39 | 40.72 |  |  |  |  |
| 4 | 7891.36 | -3882.02 | 0.844 | 6.87 | 34.36 | 43.81 | 14.95 |  |  |  |
| 5 | 7783.49 | -3812.17 | 0.806 | 29.90 | 15.64 | 34.54 | 13.40 | 6.53 |  |  |
| 6 | 7690.48 | -3749.74 | 0.782 | 26.46 | 11.68 | 14.43 | 5.50 | 15.64 | 26.29 |  |
| 7 | 7480.72 | -3628.95 | 0.810 | 16.67 | 20.96 | 6.70 | 27.32 | 12.03 | 14.78 | 1.55 |
| 3 knots | | | | | | | | | | |
| 1 | 9908.59 | -4935.20 | 1.000 | 100.00 |  |  |  |  |  |  |
| 2 | 8775.90 | -4349.75 | 0.812 | 39.86 | 60.14 |  |  |  |  |  |
| 3 | 8163.00 | -4024.20 | 0.863 | 51.72 | 7.39 | 40.89 |  |  |  |  |
| 4 | 7890.30 | -3868.75 | 0.845 | 34.36 | 14.60 | 44.16 | 6.87 |  |  |  |
| 5 | 7783.32 | -3796.16 | 0.806 | 6.53 | 34.88 | 13.92 | 15.46 | 29.21 |  |  |
| 6 | 7576.94 | -3673.87 | 0.835 | 14.26 | 15.12 | 1.37 | 6.53 | 33.85 | 28.87 |  |
| 7 | 7479.88 | -3606.24 | 0.812 | 18.21 | 27.84 | 20.27 | 11.68 | 1.55 | 13.57 | 6.87 |
| 4 knots | | | | | | | | | | |
| 1 | 9911.30 | -4933.37 | 1.000 | 100.00 |  |  |  |  |  |  |
| 2 | 8782.80 | -4346.83 | 0.812 | 59.97 | 40.03 |  |  |  |  |  |
| 3 | 8170.70 | -4018.50 | 0.864 | 40.89 | 51.72 | 7.39 |  |  |  |  |
| 4 | 7902.64 | -3862.19 | 0.846 | 34.88 | 6.87 | 43.81 | 14.43 |  |  |  |
| 5 | 7799.69 | -3788.43 | 0.806 | 34.19 | 6.53 | 14.09 | 29.55 | 15.64 |  |  |
| 6 | 7713.83 | -3723.22 | 0.785 | 26.98 | 14.95 | 11.51 | 5.67 | 15.98 | 24.91 |  |
| 7 | 7498.94 | -3593.49 | 0.815 | 28.18 | 1.37 | 18.21 | 19.76 | 7.04 | 11.68 | 13.75 |

**
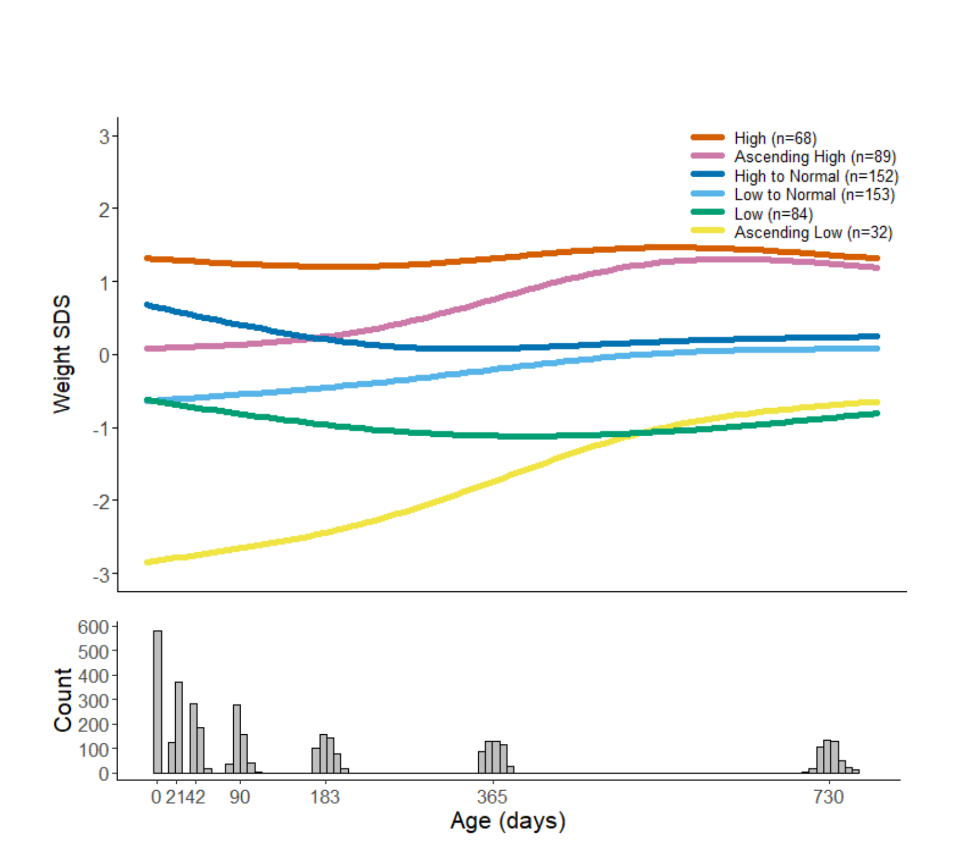
**

**Figure 1 Trajectories for latent class growth analysis six class model of weight standard deviation scores (SDS) from birth to 2 years.**

**
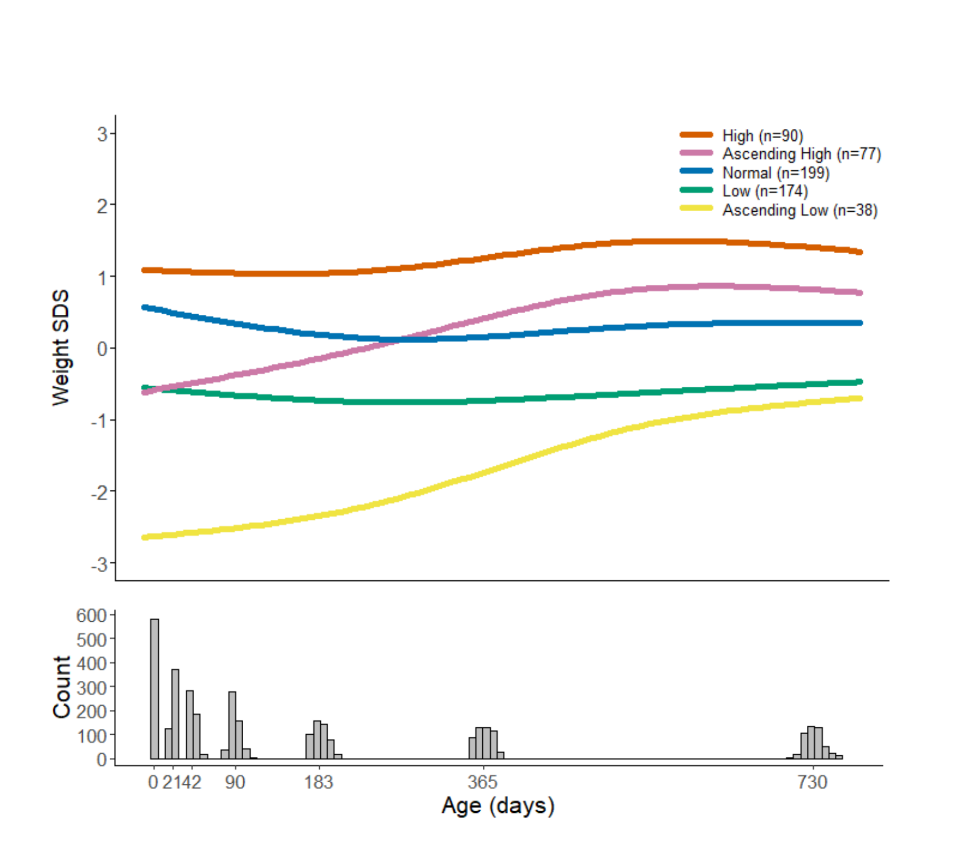
**

**Figure 2 Trajectories for latent class growth analysis five class model of weight standard deviation scores (SDS) from birth to 2 years.**

***
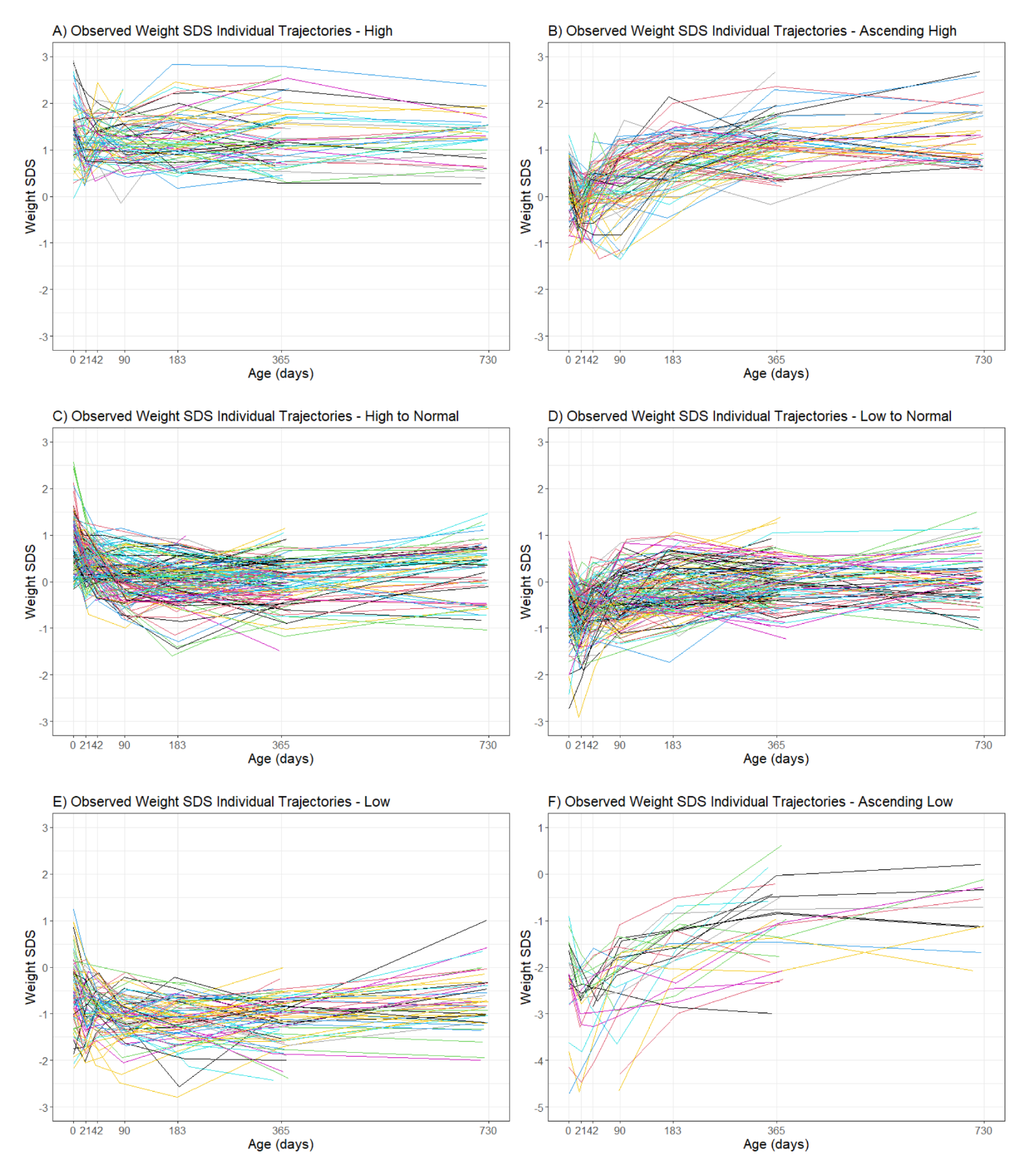
*Figure 3 Individual trajectories for latent class growth analysis six class model of weight standard deviation scores (SDS) from birth to 2 years.**

***
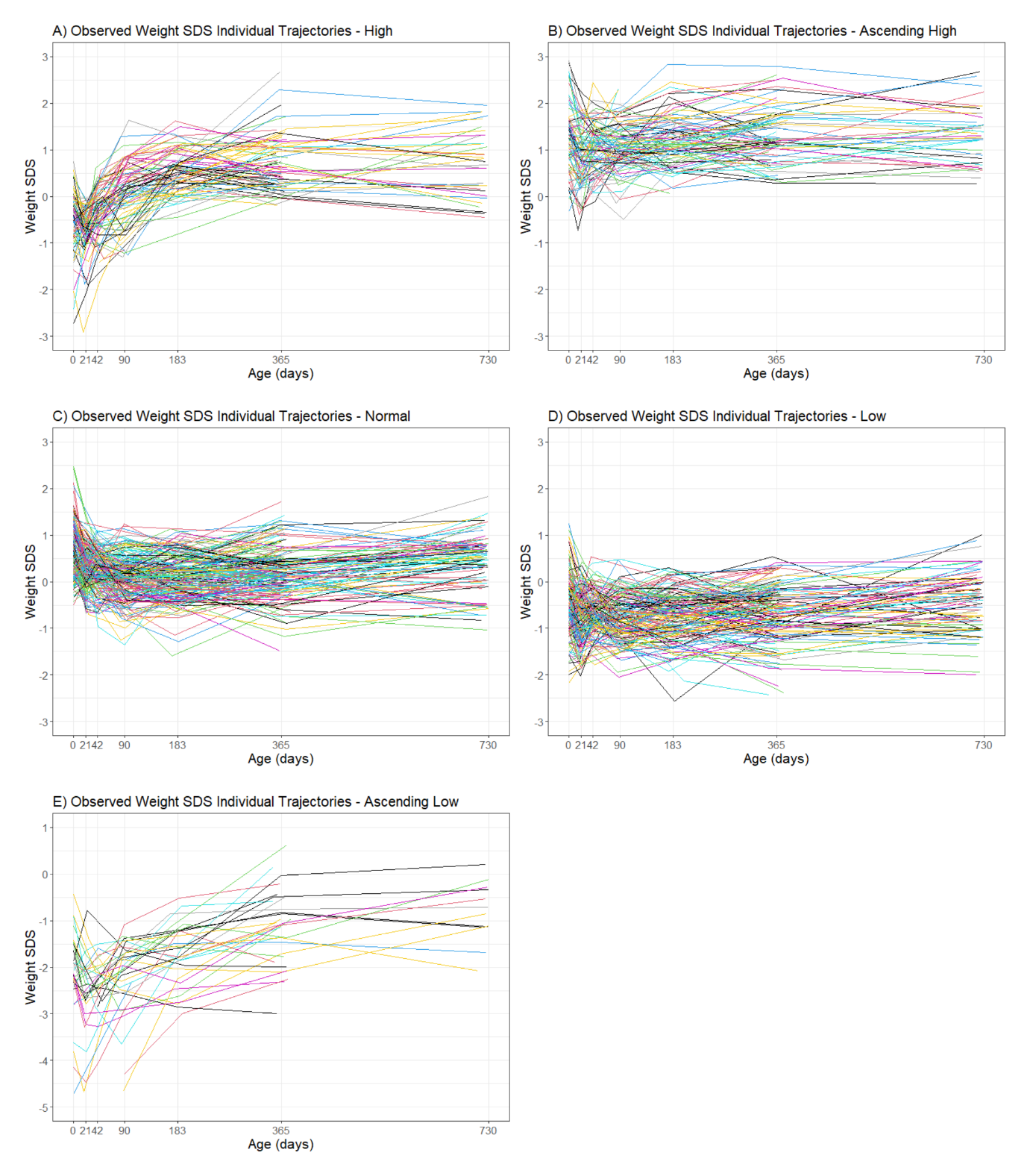
*Figure 4 Individual trajectories for latent class growth analysis five class model of weight standard deviation scores (SDS) from birth to 2 years.**

**Table 2 Latent class growth analysis model summary statistics for weight standard deviation scores (SDS) from birth to 2 years including only offspring with ≥3 visits.**

|  | BIC | Log Likelihood | Entropy | Class 1 | Class 2 | Class 3 | Class 4 | Class 5 | Class 6 | Class 7 |
| --- | --- | --- | --- | --- | --- | --- | --- | --- | --- | --- |
| 2 knots | | | | | | | | | | |
| 1 | 9680.72 | -4824.57 | 1.000 | 100.00 |  |  |  |  |  |  |
| 2 | 8520.04 | -4228.45 | 0.833 | 58.70 | 41.30 |  |  |  |  |  |
| 3 | 7960.61 | -3932.95 | 0.873 | 7.61 | 40.40 | 51.99 |  |  |  |  |
| 4 | 7675.77 | -3774.75 | 0.863 | 6.34 | 42.93 | 34.96 | 15.76 |  |  |  |
| 5 | 7555.02 | -3698.59 | 0.829 | 6.34 | 16.49 | 33.33 | 14.67 | 29.17 |  |  |
| 6 | 7464.35 | -3637.47 | 0.804 | 15.76 | 24.28 | 15.94 | 12.50 | 5.62 | 25.91 |  |
| 7 | 7338.93 | -3558.98 | 0.827 | 16.67 | 15.40 | 5.98 | 21.74 | 12.50 | 1.45 | 26.27 |
| 3 knots | | | | | | | | | | |
| 1 | 9671.59 | -4816.86 | 1.000 | 100.00 |  |  |  |  |  |  |
| 2 | 8510.43 | -4217.33 | 0.833 | 41.49 | 58.51 |  |  |  |  |  |
| 3 | 7950.76 | -3918.56 | 0.874 | 40.22 | 52.36 | 7.43 |  |  |  |  |
| 4 | 7668.85 | -3758.66 | 0.863 | 35.51 | 6.34 | 42.57 | 15.58 |  |  |  |
| 5 | 7548.57 | -3679.58 | 0.829 | 28.99 | 16.30 | 33.33 | 6.34 | 15.04 |  |  |
| 6 | 7461.33 | -3617.02 | 0.806 | 26.27 | 16.85 | 5.62 | 24.09 | 12.14 | 15.04 |  |
| 7 | 7411.89 | -3573.36 | 0.796 | 4.89 | 11.78 | 15.76 | 7.79 | 20.11 | 16.67 | 23.01 |
| 4 knots | | | | | | | | | | |
| 1 | 9672.96 | -4814.38 | 1.000 | 100.00 |  |  |  |  |  |  |
| 2 | 8515.38 | -4213.50 | 0.833 | 41.67 | 58.33 |  |  |  |  |  |
| 3 | 7957.35 | -3912.38 | 0.875 | 52.17 | 7.25 | 40.58 |  |  |  |  |
| 4 | 7679.47 | -3751.34 | 0.864 | 6.34 | 42.93 | 15.04 | 35.69 |  |  |  |
| 5 | 7562.81 | -3670.92 | 0.829 | 28.80 | 14.67 | 6.34 | 16.12 | 34.06 |  |  |
| 6 | 7476.88 | -3605.85 | 0.808 | 26.81 | 15.04 | 12.14 | 23.37 | 17.03 | 5.62 |  |
| 7 | 7417.79 | -3554.22 | 0.817 | 13.04 | 20.65 | 27.54 | 3.99 | 11.96 | 16.12 | 6.70 |

**
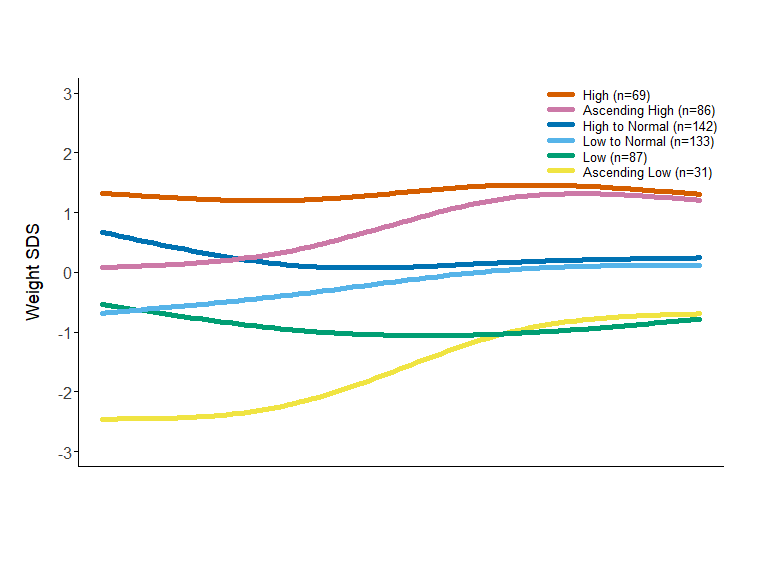
**

**Figure 5 Sensitivity analysis trajectories for latent class growth analysis six class model for weight standard deviation scores (SDS) from birth to 2 years including only offspring with ≥3 visits.**

**
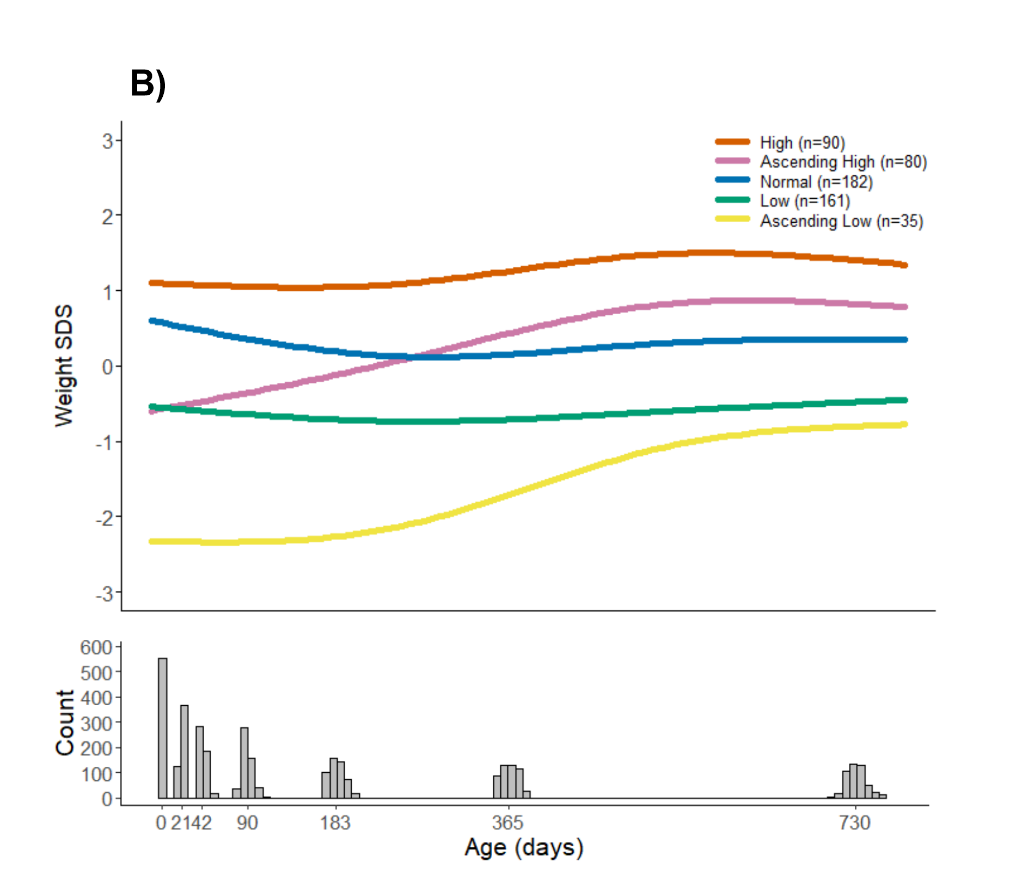
**

**Figure 6 Sensitivity analysis trajectories for latent class growth analysis five class model for weight standard deviation scores (SDS) from birth to 2 years including only offspring with ≥3 visits.**

**
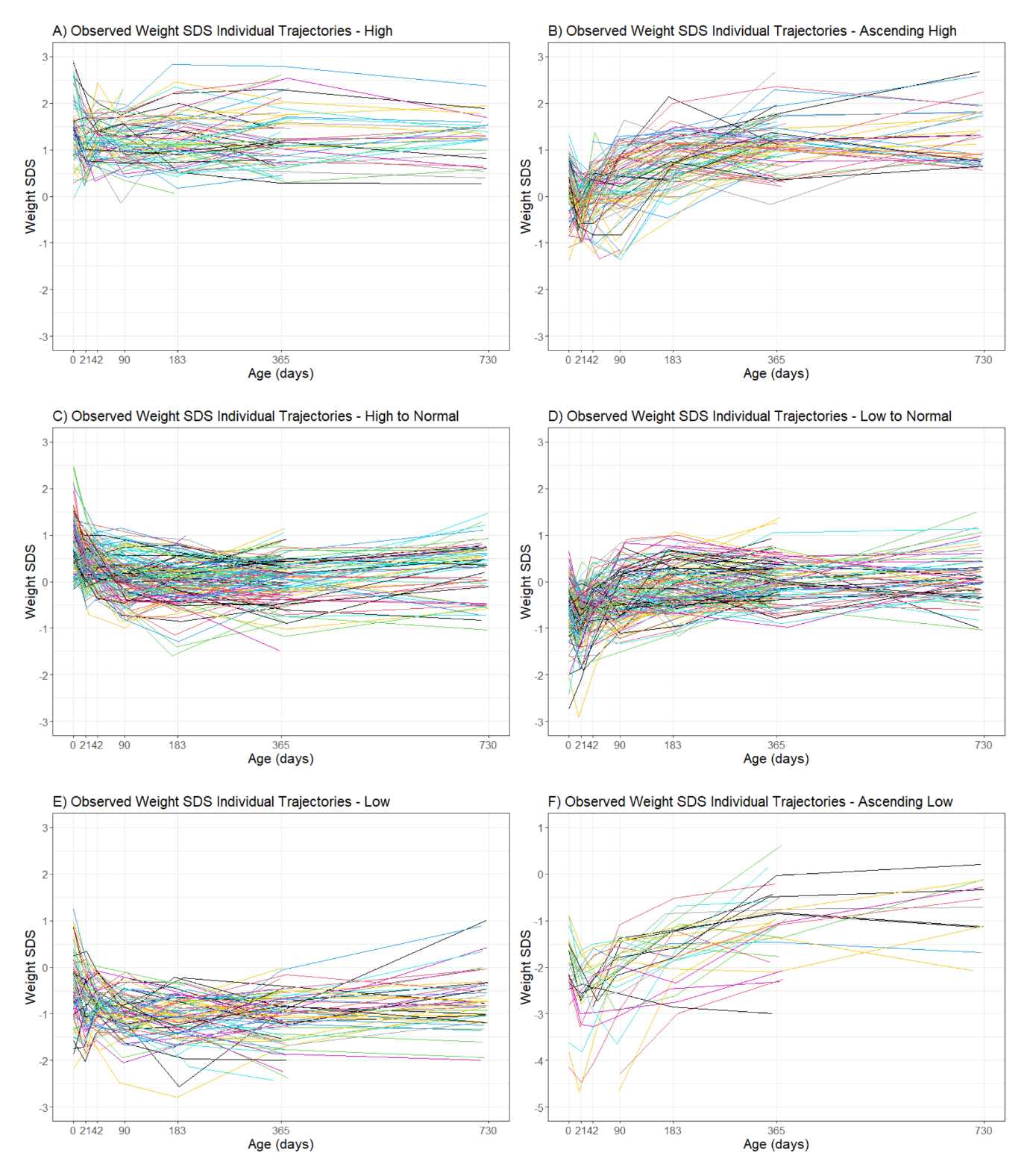
Figure 7 Sensitivity analysis individual trajectories for latent class growth analysis six class model for weight standard deviation scores (SDS) from birth to 2 years including only offspring with ≥3 visits.**

**
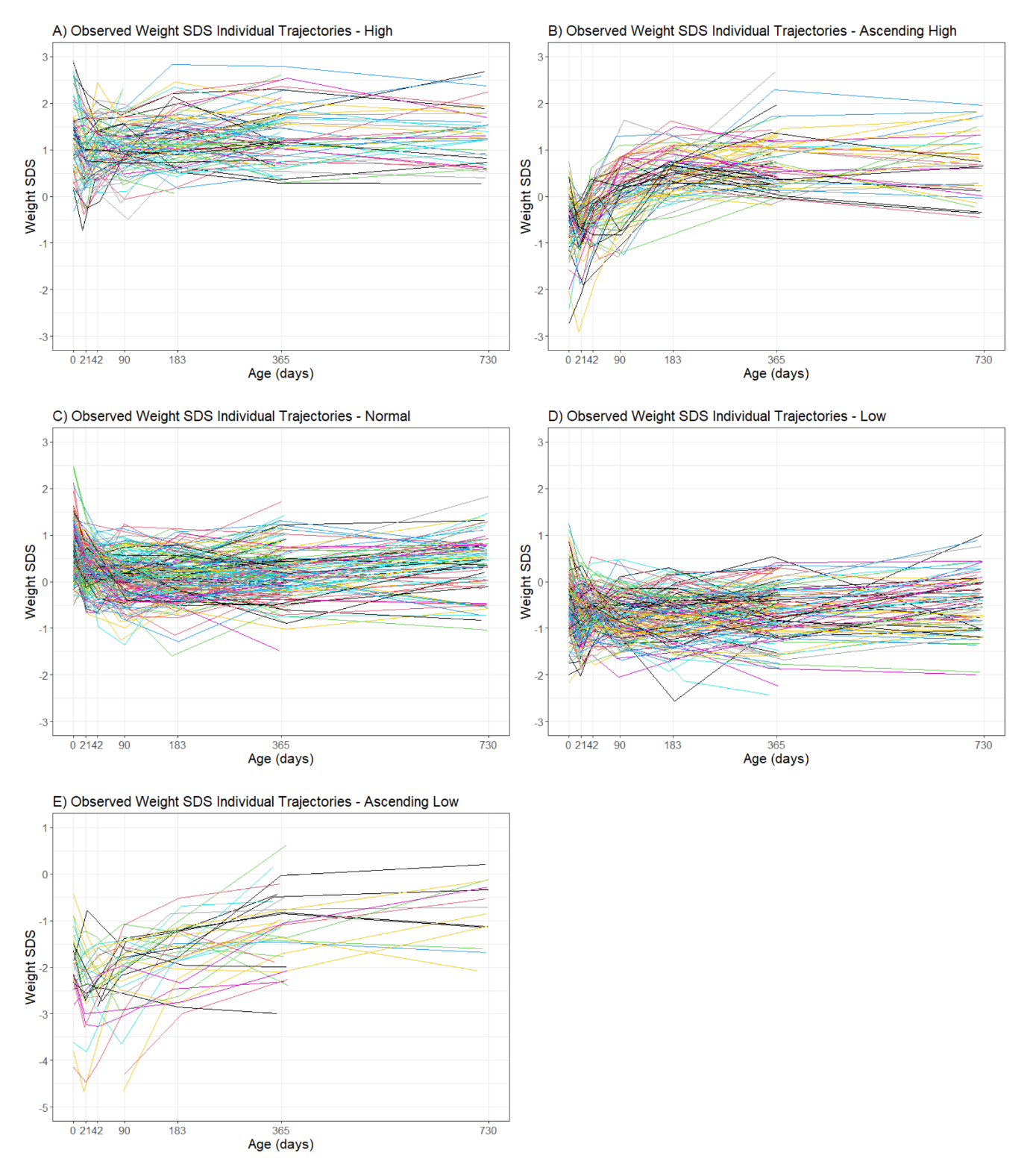
Figure 8 Sensitivity analysis individual trajectories for latent class growth analysis five class model for weight standard deviation scores (SDS) from birth to 2 years including only offspring with ≥3 visits.**

**Table 3 Risk ratios and 95% confidence intervals for the weight standard deviation scores (SDS) trajectories from the sensitivity analysis five class latent class growth analysis model.**

|  | *n* (%) | | RR (95% CI) | *p* |
| --- | --- | --- | --- | --- |
|  | Intervention | Control |  |  |
| Normal | 103 (37.5%) | 79 (28.9%) | 1.29 (1.02, 1.65) | **0.035** |
| High | 37 (13.5%) | 53 (19.4%) | 0.69 (0.47, 1.02) | 0.061 |
| Low | 90 (32.7%) | 71 (26.0%) | 1.26 (0.97, 1.64) | 0.085 |
| Ascending high | 35 (12.7%) | 45 (16.5%) | 0.77 (0.51, 1.16) | 0.216 |
| Ascending low | 10 (3.6%) | 25 (9.2%) | 0.40 (0.19, 0.81) | **0.008** |
| Data are risk ratios and respective 95% confidence intervals from logistic regression. | | | | |
