# Supplementary material for: Impact of preconception and antenatal supplementation with myo-inositol, probiotics, and micronutrients on offspring BMI and weight gain over the first 2 years
Source: BMC Med. 2024 Jan 30;22:39. doi: 10.1186/s12916-024-03246-w (PMC10826220; doi:10.1186/s12916-024-03246-w)
Supplement: Supplementary file 2 — Additional file 2: Additional cohort descriptive statistics and results from sensitivity analyses. Table S1. Number of anthropometric measurements at each visit by randomisation group. Table S2. Characteristics of the study population who provided data at 2 years. Table S3. Body mass index (BMI), weight, and length standard deviation scores (SDS) at 2 years among offspring, according to randomisation group (n = 481), with adjustment for breastfeeding duration. Table S4. Adjusted risk ratios and 95% confidence intervals of body mass index (BMI) at 2 years and rapid weight gain from birth to 1 or 2 years among sub-groups of different maternal metabolic risk. Fig. S1. Least squares means of A) body mass index (BMI), B) weight, and C) length standard deviation scores (SDS) by visit from repeated measures linear mixed models among offspring with measurements at each visit (n = 201). Figure shows the adjusted means and 95% confidence intervals for intervention (red) and control (black) offspring. *p <0.05. [file 12916_2024_3246_MOESM2_ESM.docx]

**Impact of preconception and antenatal supplementation with *myo*-inositol, probiotics and micronutrients on offspring BMI and weight gain over the first two years**

Jaz Lyons-Reid^1^, José G. B. Derraik^1,2,3,4^, Timothy Kenealy^1,5^, Benjamin B. Albert^1^, J. Manuel Ramos Nieves^6^, Cathriona R. Monnard^6^, Phil Titcombe^7^, Heidi Nield^7^, Sheila J. Barton^7^, Sarah El-Heis^7,8^, Elizabeth Tham^9,10,11^, Keith M. Godfrey^7,8,#^, Shiao-Yng Chan^9,10,11,#^, Wayne S. Cutfield^1,12,#,*^ and the NiPPeR Study Group^‡^

^1^Liggins Institute, The University of Auckland, Auckland, New Zealand

^2^Department of Paediatrics: Child and Youth Health, Faculty of Medical and Health Sciences, The University of Auckland, Auckland, New Zealand

^3^Environmental-Occupational Health Sciences and Non-communicable Diseases Research Group, Research Institute for Health Sciences, Chiang Mai University, Chiang Mai, Thailand

^4^Department of Women's and Children's Health, Uppsala University, Uppsala, Sweden

^5^Department of Medicine and Department of General Practice and Primary Health Care, The University of Auckland, Auckland, New Zealand

^6^Nestlé Institute of Health Sciences, Nestlé Research, Société des Produits Nestlé S.A., Lausanne, Switzerland

^7^MRC Lifecourse Epidemiology Centre, University of Southampton, Southampton, United Kingdom

^8^NIHR Southampton Biomedical Research Centre, University of Southampton and University Hospital Southampton NHS Foundation Trust, Southampton, United Kingdom

^9^Singapore Institute for Clinical Sciences, Agency for Science, Technology and Research (A*STAR), Singapore

^10^Human Potential Translational Research Programme, Yong Loo Lin School of Medicine, National University of Singapore, Singapore

^11^Department of Obstetrics & Gynaecology, National University of Singapore, Singapore

^12^A Better Start – National Science Challenge, The University of Auckland, Auckland, New Zealand

Corresponding author: Wayne S. Cutfield, MD, Liggins Institute, University of Auckland, Private Bag 92019, Auckland, New Zealand. Email: w.cutfield@auckland.ac.nz. Ph: +64 9 923 4476

#These authors share joint last authorship.

‡Ryan Carvalho (ryan.carvalho@nestle.com), Julie Ann Castro (julie_castro@nuhs.edu.sg), Mary Cavanagh (m.cavanagh@auckland.ac.nz), Hsin Fang Chang (hsin_fang_chang@nuhs.edu.sg), Yap Seng Chong (obgcys@nus.edu.sg), Paula Costello (pc@mrc.soton.ac.uk), Vanessa Cox (vac@mrc.soton.ac.uk), Sevasti Galani (sevasti.galani@ucl.ac.uk), Judith Hammond (j.hammond@auckland.ac.nz), Nicholas C Harvey (nch@mrc.soton.ac.uk), Soo Min Han (clara.han@auckland.ac.nz), Mrunalini Jagtap (mrunalini.jagtap1@gmail.com), Chiara Nembrini (Chiara.Nembrini@rdls.nestle.com), Justin M O’Sullivan (justin.osullivan@auckland.ac.nz), Judith Ong (judith_ong@nuhs.edu.sg), Irma Silva-Zolezzi (irma.silvazolezzi@nestle.com), Wendy Sim (sin_nie_sim@nuhs.edu.sg), Vicky Tay (Vicky_tay@sics.a-star.edu.sg), Mya-Thway Tint (Mya_Thway_Tint@sics.a-star.edu.sg), Mark Vickers (m.vickers@auckland.ac.nz), Jui-Tsung Wong (csd3589@yahoo.com), Gladys Woon (gladys_woon@nuhs.edu.sg), Wen Lun Yuan (wenlun.yuan@inserm.fr)

**Table 1 Number of anthropometric measurements at each visit by randomisation group.**

|  | Weight | | | Length^^^ | | | BMI | | |
| --- | --- | --- | --- | --- | --- | --- | --- | --- | --- |
|  | Intervention | Control | All | Intervention | Control | All | Intervention | Control | All |
| Birth | 287 | 289 | 576 | 234 | 246 | 480 | 234 | 246 | 480 |
| 3 weeks | 252 | 252 | 504 | 235 | 239 | 474 | 227 | 231 | 458 |
| 6 weeks | 253 | 244 | 497 | 242 | 233 | 475 | 239 | 230 | 469 |
| 3 months | 264 | 262 | 526 | 255 | 249 | 504 | 255 | 248 | 503 |
| 6 months | 263 | 260 | 523 | 248 | 252 | 500 | 248 | 251 | 499 |
| 1 year | 265 | 257 | 522 | 251 | 240 | 491 | 251 | 240 | 491 |
| 2 years | 246 | 251 | 497 | 243 | 247 | 490 | 239 | 245 | 484 |
| ^Includes 132 adjusted lengths at birth (intervention n=61; control n=71). | | | | | | | | | |

**Table 2 Characteristics of the study population who provided data at 2 years.**

|  | Data available  (n=484) | No data  (n=92) |
| --- | --- | --- |
| Randomisation group |  |  |
| Intervention | 239 (49.4%) | 48 (52.2%) |
| Control | 245 (50.6%) | 44 (47.8%) |
| Study site |  |  |
| UK | 136 (28.1%) | 51 (55.4%) |
| Singapore | 141 (29.1%) | 24 (26.1%) |
| New Zealand | 207 (42.8%) | 17 (18.5%) |
| Maternal ethnicity^^^ |  |  |
| White Caucasian | 285 (58.9%) | 56 (60.9%) |
| Chinese | 130 (26.9%) | 13 (14.1%) |
| South Asian | 20 (4.1%) | 11 (12.0%) |
| Malay | 18 (3.7%) | 5 (5.4%) |
| Other | 31 (6.4%) | 7 (7.6%) |
| Maternal BMI (kg/m^2^) ^‡^ | 24.8 ± 5.3 | 25.1 ± 6.3 |
| Maternal height (cm) | 164.2 ± 6.8 | 163.8 ± 6.9 |
| Parity^$^ |  |  |
| Nulliparous | 317 (65.5%) | 50 (54.3%) |
| Multiparous | 167 (34.5%) | 42 (45.7%) |
| Maternal smoking during pregnancy |  |  |
| None | 412 (85.1%) | 78 (84.8%) |
| Passive smoking | 59 (12.2%) | 8 (8.7%) |
| Active smoking | 13 (2.7%) | 6 (6.5%) |
| Infant sex |  |  |
| Male | 226 (46.7%) | 42 (45.7%) |
| Female | 258 (53.3%) | 50 (54.3%) |
| Gestational age (weeks)^*^ | 39.3 ± 1.5 | 38.9 ± 2.2 |
| Preterm | 31 (6.4%) | 11 (12.0%) |
| Term | 449 (92.8%) | 80 (87.0%) |
| Post-term | 4 (0.8%) | 1 (1.1%) |
| Birthweight (g) | 3340 ± 518 | 3256 ± 607 |
| Birthweight SDS^#^ | -0.02 ± 0.94 | 0.00 ± 0.86 |
| SGA | 39 (8.1%) | 4 (4.3%) |
| AGA | 410 (84.7%) | 80 (87.0%) |
| LGA | 35 (7.2%) | 8 (8.7%) |
| Abbreviations: SGA, small-for-gestational-age; AGA, appropriate-for-gestational-age; LGA, large-for-gestational-age; BMI, body mass index. Data are mean ± SD or *n* (%). ^South Asian includes Indian, Pakistani, and Bangladeshi mothers; Other includes mothers of mixed, Black, or Polynesian ethnicity. $Multiparous includes mothers with one or more births >24 weeks of gestation. *Preterm defined as birth prior to 37^0/7^ weeks of completed gestation, term as birth between 37^0/7^ and 41^6/7^ weeks of completed gestation, and post-term as birth at or beyond 42^0/7^ weeks of completed gestation. ^#^Calculated using the UK–WHO reference; SGA defined as below the 10th percentile (-1.282 SD) and LGA as above the 90th percentile (1.282 SD). | | |

**Table 3 Body mass index (BMI), weight, and length standard deviation scores (SDS) at 2 years among offspring, according to randomisation group (n=481), with adjustment for breastfeeding duration.**

|  | Intervention  n=238 | Control  n=243 | aMD | *p* |
| --- | --- | --- | --- | --- |
| BMI SDS | 0.53 (0.38, 0.68) | 0.65 (0.51, 0.79) | -0.12 (-0.28, 0.04) | 0.15 |
| Weight SDS | 0.17 (0.02, 0.31) | 0.25 (0.11, 0.39) | -0.08 (-0.25, 0.08) | 0.31 |
| Length SDS | -0.33 (-0.48, -0.17) | -0.25 (-0.39, -0.10) | -0.08 (-0.25, 0.09) | 0.35 |
|  | Intervention  n (%) | Control  n (%) | aRR | *p* |
| BMI >95^th^ percentile | 22 (9.2%) | 44 (18.1%) | 0.53 (0.33, 0.86) | **0.010** |
| Abbreviations: aMD, adjusted mean difference; aRR,adjusted risk ratio; SDS, standard deviation scores. Data are least squares means (i.e. adjusted means) or adjusted risk ratios and respective 95% confidence intervals from general linear models or logistic regression adjusted for study site (UK/Singapore/New Zealand), infant sex (male/female), parity (nulliparous/multiparous), maternal smoking (none/active or passive), maternal pre-pregnancy BMI (for BMI SDS and weight SDS) or maternal height (for length SDS), gestational age at birth, duration of exclusive breastfeeding (weeks), and duration of any breastfeeding (weeks). | | | | |


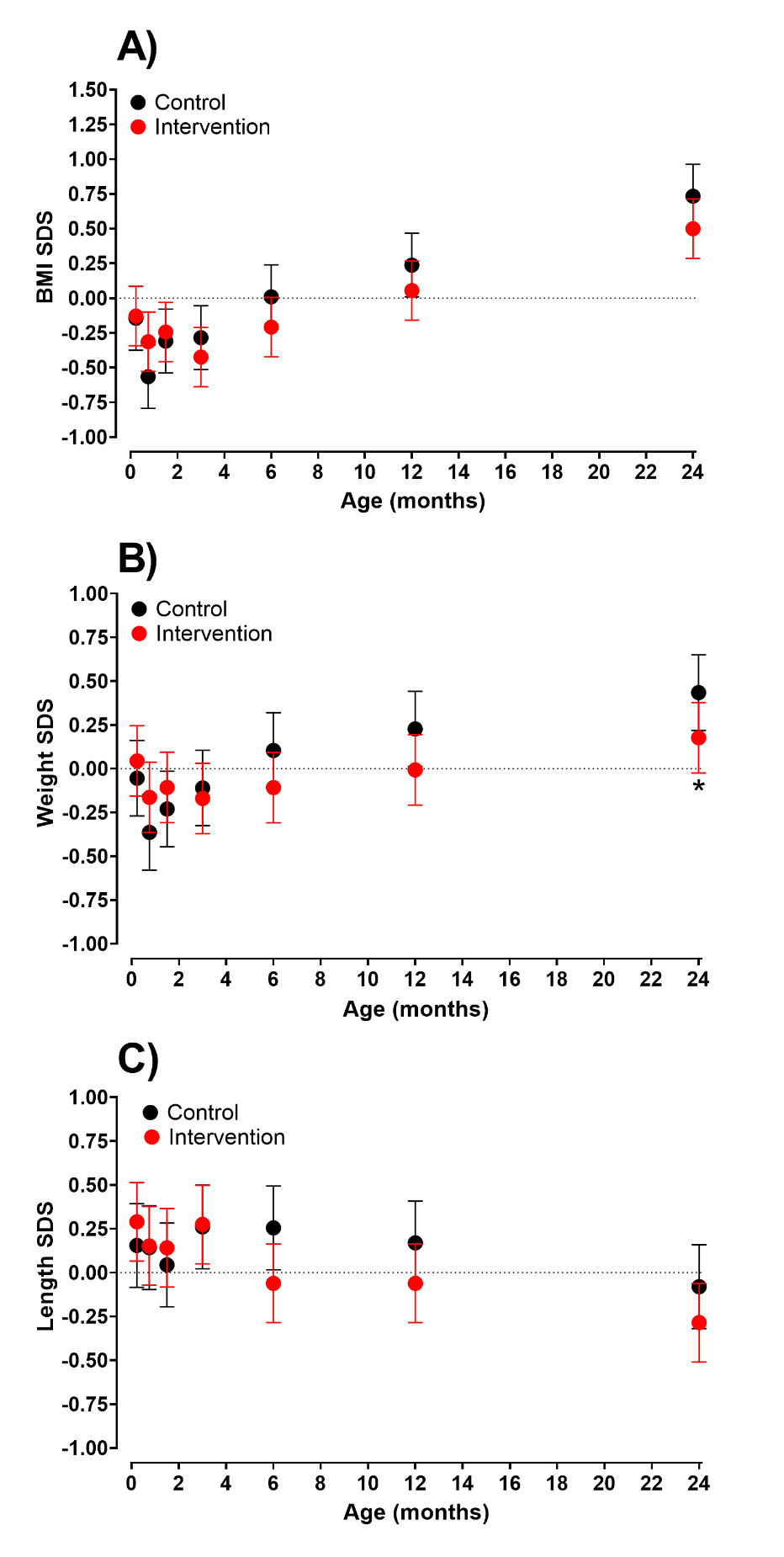


**Figure 1 Least squares means of A) body mass index (BMI), B) weight, and C) length standard deviation scores (SDS) by visit from repeated measures linear mixed models among offspring with measurements at each visit (n=201). Figure shows the adjusted means and 95% confidence intervals for intervention (red) and control (black) offspring. *p<0.05**

**Table 4 Adjusted risk ratios and 95% confidence intervals of body mass index (BMI) at 2 years and rapid weight gain from birth to 1 or 2 years among sub-groups of different maternal metabolic risk.**

|  | n (%) | | aRR (95% CI) | *p* |
| --- | --- | --- | --- | --- |
|  | Intervention | Control |  |  |
| 2-year BMI >1.65 SD | | | | |
| Overweight/Obese ^‡^ | 11 (10.8%) | 20 (18.2%) | 0.59 (0.29, 1.21) | 0.147 |
| Normal weight ^‡^ | 11 (8.0%) | 24 (17.8%) | 0.43 (0.22, 0.85) | **0.014** |
| Overweight/Obese WHO^%^ | 9 (11.7%) | 18 (18.6%) | 0.58 (0.27, 1.25) | 0.165 |
| Normal weight WHO^%^ | 13 (8.0%) | 26 (17.6%) | 0.42 (0.22, 0.81) | **0.009** |
| GDM^#^ | 4 (6.9%) | 13 (23.2%) | 0.26 (0.08, 0.86) | **0.028** |
| No GDM^#^ | 18 (10.0%) | 30 (16.5%) | 0.61 (0.35, 1.05) | **0.073** |
| >0.67 SD from birth to 1 year | | | | |
| Overweight/Obese | 30 (26.1%) | 36 (32.1%) | 0.86 (0.58, 1.27) | 0.448 |
| Normal weight | 28 (18.7%) | 44 (30.3%) | 0.63 (0.43, 0.93) | **0.019** |
| Overweight/Obese WHO | 14 (16.1%) | 32 (32.7%) | 0.68 (0.39, 1.18) | 0.170 |
| Normal weight WHO | 44 (24.7%) | 48 (30.2%) | 0.74 (0.53, 1.02) | **0.069** |
| GDM | 18 (27.7%) | 25 (43.1%) | 0.54 (0.33, 0.88) | **0.014** |
| No GDM | 40 (20.1%) | 54 (28.1%) | 0.86 (0.61, 1.20) | 0.367 |
| >1.34 SD from birth to 2 year | | | | |
| Overweight/Obese | 9 (8.6%) | 21 (18.4%) | 0.54 (0.28, 1.04) | 0.066 |
| Normal weight | 10 (7.1%) | 22 (16.1%) | 0.52 (0.26, 1.02) | 0.056 |
| Overweight/Obese WHO | 3 (3.8%) | 19 (19.0%) | 0.31 (0.09, 1.03) | 0.056 |
| Normal weight WHO | 16 (9.6%) | 24 (15.9%) | 0.52 (0.29, 0.96) | **0.035** |
| GDM | 5 (8.3%) | 11 (19.3%) | 0.29 (0.13, 0.67) | **0.004** |
| No GDM | 14 (7.6%) | 31 (16.6%) | 0.66 (0.37, 1.18) | 0.157 |
| Abbreviations: aRR, adjusted risk ratio; CI, confidence interval; GDM, gestational diabetes mellitus. Data are adjusted risk ratios and respective 95% confidence intervals from logistic regression adjusted for study site (UK/Singapore/New Zealand), infant sex (male/female), parity (nulliparous/multiparous), maternal smoking (none/active or passive), maternal pre-pregnancy BMI, and gestational age at birth. Statistically significant comparisons (*p*<0.05) are shown in bold. ‡Defined using ethnic-specific thresholds for overweight and obesity: BMI ≥23 to <27.5 and ≥27.5 kg/m^2^, respectively, for Asians, including Chinese, Indians, Pakistani, Bangladeshi, Malay, mixed Asian; BMI ≥25 to <30 and ≥30 kg/m^2^, respectively, for non-Asians, including White Caucasian, Polynesian, Black, mixed Asian-non-Asian. %Defined using the WHO cut-points for overweight and obesity: BMI ≥25 to <30 and ≥30 kg/m^2^. #Defined using the International Association of Diabetes and Pregnancy Study Groups criteria (fasting glucose ≥5.1 mmol/L or 1-h glucose ≥10.0 mmol/L or 2-h glucose ≥8.5 mmol/L). | | | | |
